# Using *in silico *models to simulate dual perturbation experiments: procedure development and interpretation of outcomes

**DOI:** 10.1186/1752-0509-3-44

**Published:** 2009-04-30

**Authors:** Neema Jamshidi, Bernhard O Palsson

**Affiliations:** 1 Department of Bioengineering, 9500 Gilman Drive, University of California, San Diego, La Jolla, CA 92093-0412, USA

## Abstract

**Background:**

A growing number of realistic *in silico *models of metabolic functions are being formulated and can serve as 'dry lab' platforms to prototype and simulate experiments before they are performed. For example, dual perturbation experiments that vary both genetic and environmental parameters can readily be simulated *in silico*. Genetic and environmental perturbations were applied to a cell-scale model of the human erythrocyte and subsequently investigated.

**Results:**

The resulting steady state fluxes and concentrations, as well as dynamic responses to the perturbations were analyzed, yielding two important conclusions: 1) that transporters are informative about the internal states (fluxes and concentrations) of a cell and, 2) that genetic variations can disrupt the natural sequence of dynamic interactions between network components. The former arises from adjustments in energy and redox states, while the latter is a result of shifting time scales in aggregate pool formation of metabolites. These two concepts are illustrated for glucose-6 phosphate dehydrogenase (G6PD) and pyruvate kinase (PK) in the human red blood cell.

**Conclusion:**

Dual perturbation experiments *in silico *are much more informative for the characterization of functional states than single perturbations. Predictions from an experimentally validated cellular model of metabolism indicate that the measurement of cofactor precursor transport rates can inform the internal state of the cell when the external demands are altered or a causal genetic variation is introduced. Finally, genetic mutations that alter the clinical phenotype may also disrupt the 'natural' time scale hierarchy of interactions in the network.

## Background

*In silico *models of complex biological processes are now being built. The scope of such models can range from genetic circuits [1,2], to organelles [1], to whole cells [2,3], to whole organs [4]. Computational models are increasingly being recognized as important investigational tools for the analysis of complex biological systems [5]. There are now suggestions that even simulations of a whole human being may one day become possible; the virtual human [6]. Such models are being used to accelerate discovery [7,8], develop understanding of complex physiological processes [9], and for prospective biological design [10].

Some *in silico *whole cell models can now represent cellular functions mechanistically with a reasonable degree of accuracy [11,12]. Understanding and properly characterizing the function of a biological network includes characterization of how the network responds to different types of perturbations; environmental and/or genetic. Critical to eliciting the differences between two seemingly similar systems is the application of a stress on the systems, i.e. the systems need to be perturbed in order to determine whether their functional capabilities have changed. Thus, dual perturbation experiments are used to interrogate the functional abilities of cells. Just as for experimental models in biology, relative predictions by *in silico *models may be more useful than absolute predictions. This use of *in silico *models can now be prototyped at the single cell level, and *in silico *models of single cells should be used to predict outcomes of dual perturbation experiments (that cross genetic and environmental perturbations) before they are performed in the laboratory. Kinetic network models can be particularly useful for perturbation experiments, since they 1) enable predictions to be made for steady state fluxes as well as concentrations, 2) enable investigation of the dynamic properties when moving from one steady state to another, 3) allow perturbations to be made through alteration of enzyme parameters, initial conditions for concentrations, or the application of various 'load' functions or alteration of enzyme rate laws, 4) enable analysis of dynamics when moving from one steady state to another, 5) enable analysis of non-linear properties of networks.

We adopted the approach of using perturbation experiments in an effort to better understand the changes that occur at metabolic network steady states. A set of genetic variants were analyzed following environmental perturbations and compared to normal cells undergoing the same environmental perturbations. The well established cell-scale kinetic model of human red cell metabolism was used for these studies [13,14]. This network accounts for glycolysis, the pentose phosphate pathway, the Rapoport-Luebering Shunt, nucleotide salvage pathways, as well as sodium and potassium transport channels and the sodium potassium ATPase, described by 34 ODEs comprised of 44 enzyme rate expressions with allosteric influences when appropriate, in addition to magnesium complexing reactions [15]. The detailed description of the allosterically regulated enzymes, such as glucose-6 phosphate dehydrogenase (G6PD), pyruvate kinase (PK), and phosphofructokinase (PFK) enabled the direct application of causal SNP mutations to study well-known genetic and environmental variations. Since fluxes describe the functional states of cells and 'tie' the network together and metabolite concentrations are the most direct descriptions of biochemical phenotypes, we used this kinetic model of metabolism to investigate changes in fluxes as well as concentrations. It has previously been demonstrated that this experimentally validated dynamic model of red cell metabolism can be used to differentiate among phenotypes with a range of clinical severity [16]. In the present study we focused on well-known genetic variations in G6PD and PK and changes in environmental parameters that reflect changes in redox and energy loads on the cell and that these changes can be inferred based on transporter flux states and cofactor concentration ratios and without relying on extensive simulations.

## Results

The red blood cell's primary physiological function is to deliver oxygen that enters the body through the lungs, to tissues and to return carbon dioxide back to the lungs, to be expelled from the body. In order to carry out these respiratory functions, basic but critical metabolic functionality in the red cell must be maintained; including maintaining adequate ATP levels to maintain cell shape and the preservation of reduced glutathione to counteract oxidative stresses. These capabilities are directly affected to varying degrees in patients with metabolic enzyme deficiencies. The two most common of such deficiencies in the human red cell are in G6PD and PK [17,18]. We considered dual perturbations *in silico *where genetic variations in the properties of these two enzymes are crossed with changing environmental conditions.

### 1. Baseline responses of the 'normal' red cell: elucidating the role of pooled variables and transporters

**Figure 1:**
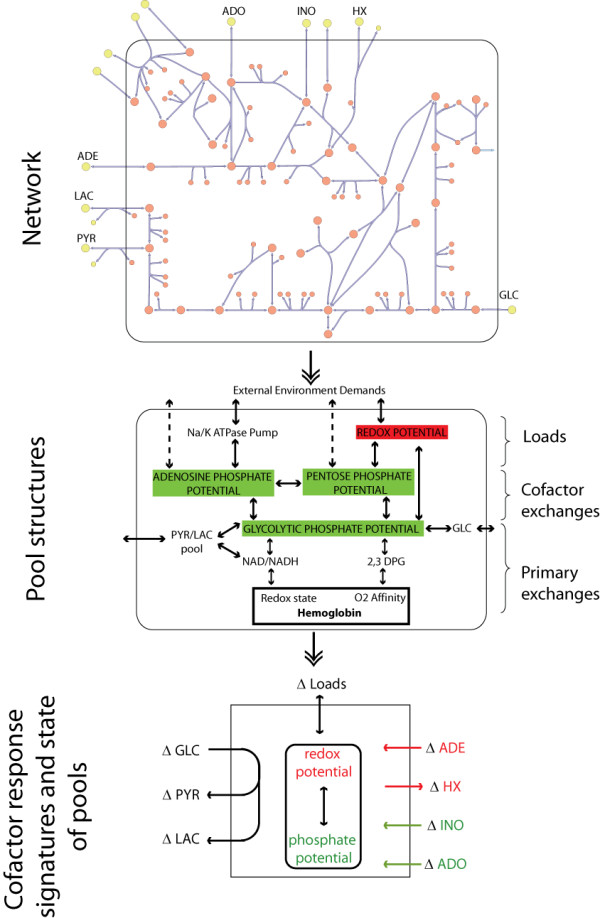
**Simplification of a network to aggregate pooled variables**. Schematic diagram of the human red cell network that includes 34 metabolites and 44 reaction fluxes. The network can be simplified into biologically relevant pooled variables and metabolite ratios, such as cofactor pools. Further simplification will result in classification of the network functional states based on changes in transport fluxes. The network is coarse-grained here in order to highlight the ability to characterize different functional states based only on the lumped aggregate variables representing physiological states (redox potential and phosphate potential) and key transporters (HX, ADE, and ADO). When the cell needs to increase its redox potential in order to counteract an oxidative stress, ADE uptake and HX secretion are increased, while ADO and INO uptake are decreased. The reverse trends occur with increased energy loads. The relative magnitudes of the changes tend to be larger with ADE and ADO than INO and HX. Abbreviations: GLC: glucose, PYR: pyruvate, LAC: lactate, Na: sodium, K: potassium, HX: hypoxanthine, INO: inosine, ADE: adenine, ADO: adenosine.

The 'normal' or 'nominal' set of parameters in the dynamic model of red cell metabolism (abbreviated here as the nRBC) formed a reference case from which parameters can be perturbed. The dynamic responses nRBC (Figure 1, top) were analyzed under conditions of increased redox and energy loads to provide a baseline, or the 'normal' response to such stresses. The nRBCunder increased redox vs. increased energy loads resulted in different flux states, notably shifting between reliance on the glycolytic pathway versus the pentose phosphate pathway. The computed steady state concentrations and fluxes are provided in Additional file 1.

The evaluation of the computed nRBC metabolic network responses and the resulting internal changes from the imposition of redox or energy loads led to the observation that changes in transport fluxes reflected changes in internal functional states (Additional file 2, Figure 1):

• As the redox load increased, flux through glycolysis decreased and flux through the pentose phosphate pathway increased. At very high redox loads, the flux through phosphoglucoisomerase (PGI) even reversed direction to achieve maximal total flux through the oxidative branch of the pentose phosphate pathway. These expected shifts in pathway uses were accompanied by some unexpected changes in transport fluxes. There was a dramatic decrease in adenosine (ADO) uptake and a moderate increase in the adenosine deaminase (ADA) flux. Hypoxanthine (HX) excretion increased and inosine (INO) uptake decreased. Internally, the fluxes through adenine phosphoribosyltransferase (ADPRT) and phosphoribosylpyrophosphate synthetase (PRPPSYN) increased significantly.

• As the energy load is increased, flux through the pentose phosphate pathway decreased and flux increased through glycolysis. The adenosine kinase (AK) flux decreased, with a relatively small decrease in AMP deaminase (AMPDA), adenine phosphoribosyltransferase (ADPRT), IMP nucleotidase (IMPase) and purine nucleoside phosphorylase (PNPase). The phosphoribosylpyrophosphate synthetase (PRPPSYN) flux decreased. These changes in the flux state change the state of transporters. HX excretion decreased and INO uptake increased slightly. There was a reduction in adenine (ADE) uptake and a parallel increase in adenosine (ADO) uptake.

Taken together, these *in silico *results showed that the ratio of the adenosine to adenine uptake reflected whether the cell was responding to an increased redox load or an energy load.

#### Interpretation

Red cell metabolic functions have previously been interpreted [18,19] in terms of pooled concentration variables and ratios thereof; such as redox and phosphate capacities and potentials (Figure 1, middle). This approach led to a functional (or physiological) description of the state of red cell metabolism, rather than a detailed biochemical description. The computations of changes in fluxes and concentrations at varying redox and energy loads in the nRBC can be interpreted within this functional network description.

The alterations in the computed internal function state of the nRBC in response to environmental parameters can be reduced to changes in the states of transport fluxes (Figure 1, bottom). Increases in energy demands will shift the internal pathways to maintain the phosphate potential and ADO uptake will increase, while ADE uptake decreases and HX secretion decreases. Conversely, when increased oxidative stresses are experienced, the fluxes shift towards the redox potential with decrease in ADO uptake and an increase in ADE uptake and HX secretion.

These simulated responses provide the baseline case for the nRBC and they can be characterized in terms of a functional description of the state of nRBC metabolism (Figure 1). The key result here was that the environmental loads shift the use of pathways and the redox/energy pools. The state of these pools (their charge = occupancy/capacity [18,19]) was adjusted by alterations in key transport fluxes that are accessible to measurement.

#### Dual perturbations *in silico*

**Figure 2:**
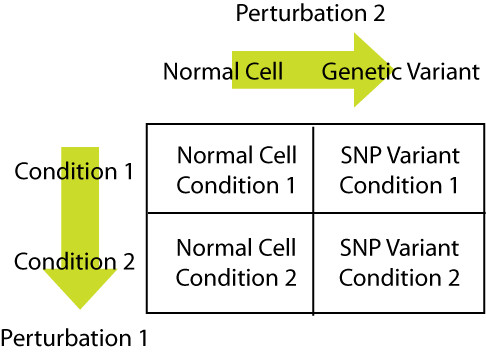
**2 × 2 design of the *in silico *dual perturbation experiments**. The condition change for the G6PD variant was an altered redox load and the condition change for the PK variant was an altered energy load.

Since the simulated flux uptake patterns reflected changes in the internal states of the nRBC to environmental perturbations, it suggested the possibility to characterize the responses different genetic variants (nRBC versus vRBC; 'v' for 'variant') in the same fashion. The responses of the nRBC were again used as a reference. This led to the notion of a dual perturbation experiment *in silico *as a way to analyze and design experiments to determine the effects of genetic variation. Differences in functional capabilities were assessed by comparisons between different states in two different environmental conditions for the nRBC vs. a vRBC (i.e. any one of the 6 G6PD variants), see Figure 2. Calculating the differences between the rows in Figure 2 and then comparing the nRBC to the vRBC resulted in a dual perturbation comparison, in which the one perturbation reflected an environmental change and the other results from a genetic mutation. Such *in silico *studies were carried out for G6PD variants and PK variants whose altered kinetic parameters were measured from patients [17-20].

**Figure 3:**
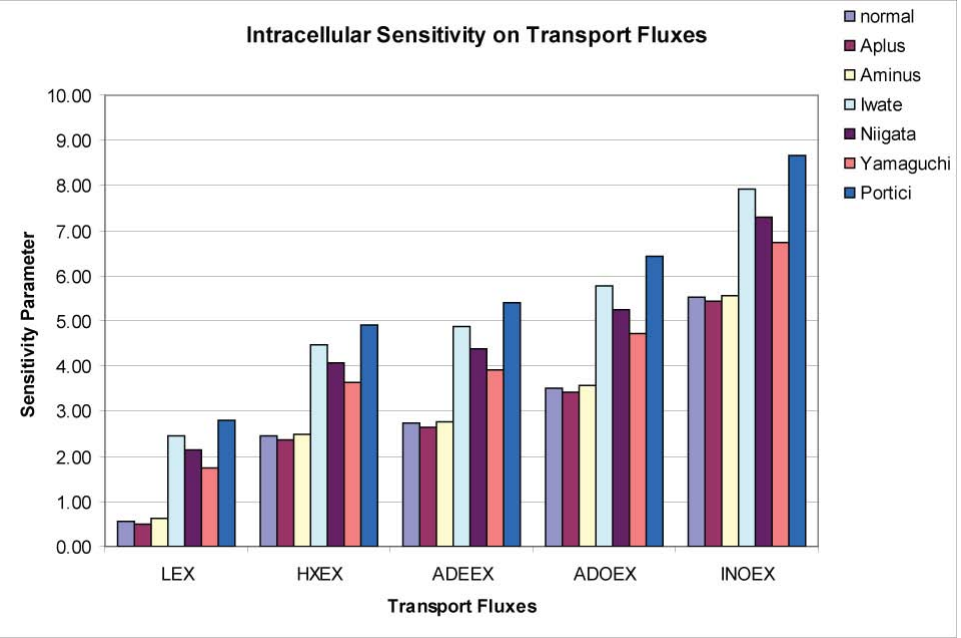
**Sensitivity of intracellular cofactor pools on extracellular transporters**. The sensitivity parameter is a logarithmic ratio between the changes in redox potential between two states over the relative change in a particular transport flux between two states. Changes in the intracellular redox state can be reflected in the extracellular transport of related metabolites.

The steady state flux distributions for the nRBC along with the six G6PD variants (vRBC_i_, i = 1, 6) were calculated at a high and a low redox load (see METHODS). The comprehensive set of steady state fluxes and concentrations calculated for the cell under different conditions led to the definition of a sensitivity parameter relating the intracellular redox state of the cell and extracellular transporters,

(1)

where R_high _and R_low _reflect the redox state of the cell (R = NADPH/(NADP + NADPH)) under high and low redox loads, respectively, and ν is a corresponding transport flux. A logarithmic sensitivity measure was used since this proved to be a sensitive relationship. The computational results illustrated how the change in the redox state of the cell (summarized as the relative size of the reduced NADP pool), an intracellular quantity, was tracked by transport fluxes, an extracellular quantity (see Figure 3). Hence, changes in the internal states of cells were detected by measuring the exchange rates.

**Figure 4:**
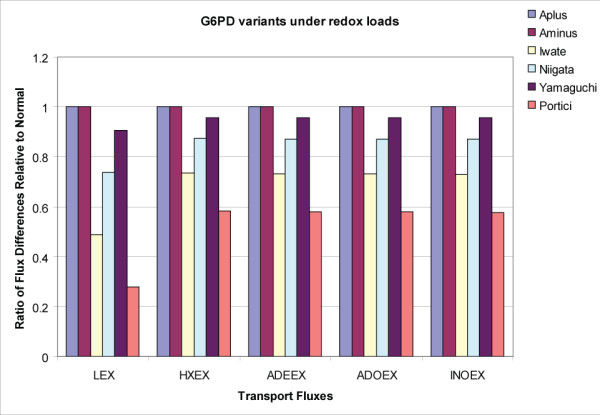
**Characterization of G6PD genetic variants as functions of transport fluxes**. The steady state flux differences were calculated for the nRBC and the vRBC variants considered at high and low redox loads for each case, respectively. The SNP variants were then normalized with respect to the flux differences for the normal case. There is a range of clinical phenotypes exhibited in these patients, determined by the severity of their hemolytic disorders. The A minus and A plus variants exhibit a more mild phenotype with non-chronic hemolytic anemia. The transport fluxes for these cases are effectively the same as the nRBC. In contrast, the other variants, with more severe phenotypes, have altered steady state flux differences compared to the normal case. Abbreviations: LEX: lactate transport, HXEX: hypoxanthine transport, INOEX: inosine transport, ADEEX: adenine transport, ADO: adenosine transport.

To further elaborate the relationship between the cofactor pools and transport fluxes in the cell, the differences between the high and low exchange fluxes were calculated for each variant and then normalized to the corresponding flux difference in the nRBC. A value of 1 indicates no difference compared to the nRBC, whereas values greater than or less than one reflect differences between the vRBCs from the nRBC. The computational results showed that the transport fluxes alone are enough to distinguish between the less severe (A+ and A-, vRBC_1 _and vRBC_2_, respectively) and more severe (Iwate, Niigata, Yamaguchi, and Portici, vRBC_3 _through vRBC_6_, respectively) SNP variants (Figure 4). This finding was interesting because it suggested that changes in transport fluxes could identify changes in internal functional states, differentiating between normal and pathophysiological situations, and that changes were identified using relative changes in the fluxes.

**Figure 5:**
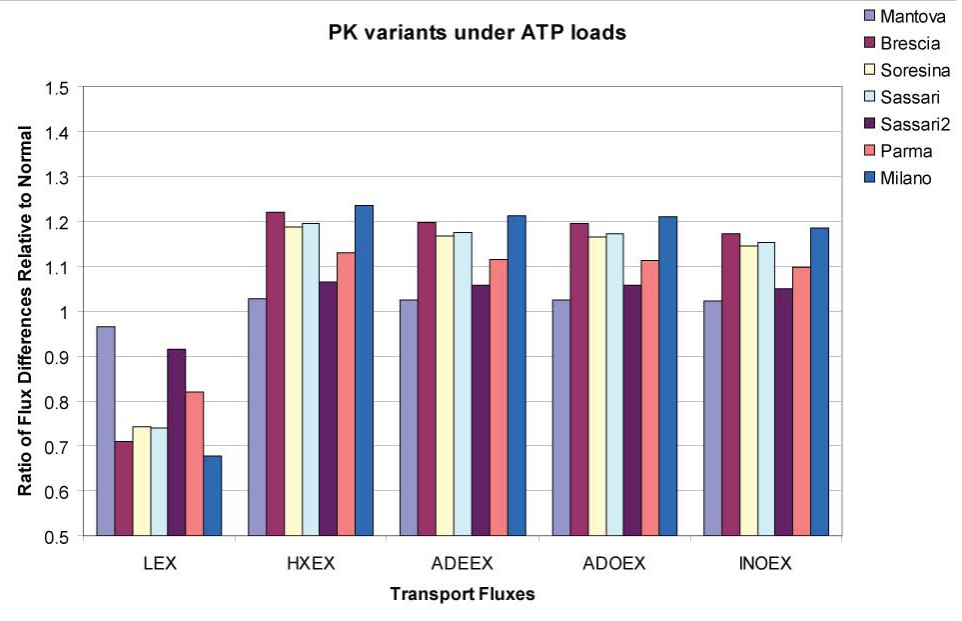
**Characterization of PK genetic variants in terms of transport fluxes**. Calculations analogous to those in Figure 2 were carried out for the PK variants with high and low energy loads. All of these variants showed altered uptake/secretion patterns for the transport fluxes compared to the normal red cell.

PK dual perturbation studies were carried out by applying different energy loads instead of redox loads (Figure 5). All of the vRBCs considered differed from the nRBC, but to varying degrees. Decreased lactate production by all of the variants reflected a decreased glycolytic flux due to the decreased activity of PK. The increased transport of the other metabolites was a result of the need to balance all carbon uptake and secretion. The Mantova variant was predicted to be less severe than the other vRBCs. In these cases, the altered transport fluxes reflected a decreased total pool size of ATP internally.

### 2. Dynamic response of vRBCs to redox loads: elucidation of altered time-scale hierarchy

In order to further characterize the differences that occur between the nRBC and the vRBCs, the dynamic states of the vRBCs were analyzed by comparing changes in the dynamic sequence of events that occur when they respond to a perturbation. Different biochemical inter-conversions, illustrated in Figure 1B, occured on different time scales [21] when the nRBC was exposed to an environmental perturbation. This sequence of events can be different in a vRBC and how much they differ *in silico *from the nRBC could be a measure of the pathological severity of the genetic variation.

#### Dynamic responses of the nRBC

The dynamic response of a network can be characterized through the comprehensive identification of the time-sequence of pool formation between the metabolites in a network [22]. This approach for determining the temporal structure in metabolic network responses was used as a basis for the analysis how it responds to perturbations. As the nRBC responded to an increased redox load, the shift in flux from glycolysis to the pentose pathway was also reflected in changes in the time scales for pooling of metabolites (see Additional file 2, Figure 1). The shifts in pooling time scales were observed throughout the network, however the most pronounced shifts involved the interactions between GL6P and the hexose-phosphates, the pentose phosphates and glutathione/NADPH, and the adenosine phosphates (AMP, ADP, ATP) with the nucleotide precursor and degradation products.

**Figure 6:**
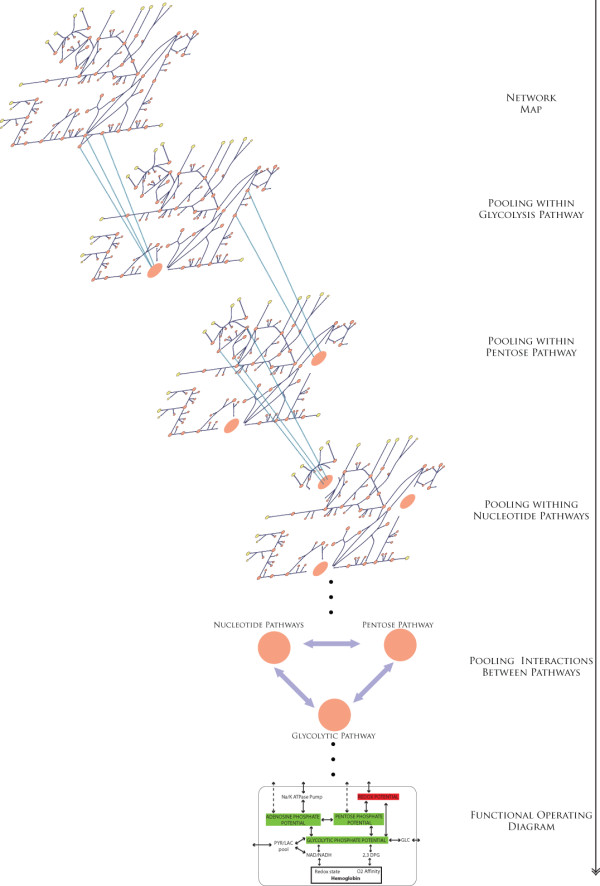
**Schematic overview of pooling interactions occurring over progressive time scales**. Generally pooling occurs within particular pathways on the fast time scales and these pools of metabolites from the different pathways then interact with one another on the slower time scales. Changes in pooling time scales that occur within pathways can affect overall interactions between larger modules in the network. The pooling interactions do not happen strictly in this manner and there are overlapping interactions between pathways over time.

Metabolite pooling over progressive time scales involves a complex series of interactions. Pool formation on fast time scales generally reflect fast achievement of equilibrium between isomers and other metabolites whose steady state concentration ratios are close to their equilibrium ratios [25,26]. Pooling on slower time scales involves interactions between aggregate pools between different pathways in the network. These interactions are illustrated in Figure 6, in order to highlight the distinction between pooling within pathways and interactions between pathways.

#### Responses of Glucose-6-Phosphate Dehydrogenase Deficient RBCs

The G6PD variants due to causal SNP mutations were analyzed in terms of their effect on the time progression of the pooling process. Modal decomposition [23] was carried out for each of these cases under low and high redox loads. The pooling between metabolites over time was then determined for the normal cell (Additional file 2, Figure 1) and compared to each of the genetic variants under high redox loads (see Additional file 2, Figures 2, 3, 4, 5, 6, 7) [22].

**Figure 7:**
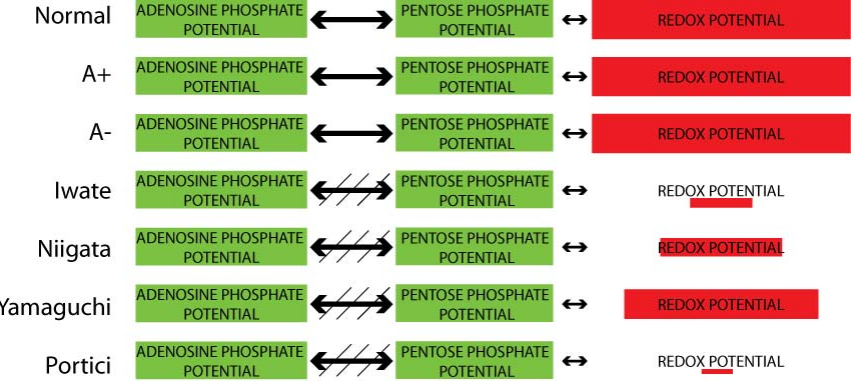
**Disruptions of the global pooling structure in G6PD variants**. The size of the boxes reflects the size of the redox and phosphate potentials. The NADPH redox state (NADPH/(NADP + NADPH)) is used as the surrogate for the overall redox potential. The A+ and A- variants are almost identical to the normal cell. The chronic hemolytic variants however have significant changes in the size of their redox pools. Additionally, the dynamics of the interactions between the adenosine and pentose phosphate pathways are disrupted in the more severe variants (slanted lines through arrows).

Changes in metabolite pooling over progressive time scales for the non-chronic hemolytic variants (A+ and A-; these patients exhibit a relatively mild form of hemolytic anemia [17]) were very similar to the normal cell (Additional file 2, Figures 1, 2, 3), with the significant differences in pooling between metabolites occurring between members of the pentose phosphate pathway and some of the nucleotide precursors. The chronic hemolytic anemia variants in contrast, exhibited more pronounced changes in the dynamic sequence of interactions. The largest differences involved; 1) interactions between the oxidative branch of the pentose phosphate pathway and the first half of glycolysis, and 2) interactions among members within the non-oxidative branch of the pentose phosphate pathway and the nucleotide salvage pathway metabolites. The alterations in dynamics of the oxidative branch in the pentose phosphate pathway resulted in disruptions of the interactions between the different pathways on slower time scales (Figure 6).

Considering network functions in the context of the functional pools described in the middle panel in Figure 1, a summary of the alterations in metabolic states under increased redox loads of the six genetic variants considered are shown in Figure 7. The areas of the boxes reflect the size of the respective metabolite pools at the steady state. It is immediately apparent that the chronic hemolytic variants have substantially reduced abilities to counteract oxidative stresses.

Additionally there were changes in the dynamics of the chronic hemolytic variants, primarily among the metabolites that contribute to the adenosine phosphate potential and the pentose phosphate potential. The last four variants exhibited altered dynamic interactions among members of the nucleotide salvage pathways and the non-oxidative branch of the pentose phosphate pathway. These changes are denoted by slanted arrows in Figure 7.

### 3. Dynamic response of the nRBC to energy loads

Similar computations of interactions among metabolites across time scales were applied to characterize the dynamic response of the nRBC to changes in energy loads (see Additional file 2, Figure 8 and METHODS). As discussed above, when the nRBC responds to an increased energy load, flux through glycolysis is increased at the cost of a reduced flux through the pentose pathway. When the dynamic pooling among metabolites for the nRBC at a normal versus an increased load were compared, there was broad shifting of pooling between metabolites, with significant changes occurring between glycolytic intermediates and adenosine metabolites, and between nucleotide precursors and pentose phosphates.

#### Pyruvate Kinase Deficient Cases

In order to characterize the time hierarchical functionality of PK genetic variants, the dynamic pooling correlations were calculated for each vRBC under high-energy load conditions. Each vRBC was then compared to the nRBC under high-energy load conditions (see Additional file 2, Figures 9–15). The salient observations from these computations were: 1) the vRBCs all exhibited comparatively similar patterns of variation from the responses of nRBC (unlike the G6PD variants considered above), and 2) the changes between high and low energy load in the variants were all similar to the changes observed for the nRBC. Since the energy charge remained fairly constant for all of the variants as well, the changes resulting from PK deficiency were reflected primarily as changes in the pool size of available ATP.

## Discussion

The utility of *in silico *models for elucidating the genotype-phenotype relationship through analysis of data from patients with two enzymopathies resulting from causal SNPs has been illustrated [16]. It has been established that intracellular cofactor ratios reflect intracellular functional states [24,25]. Cofactor secretion has previously been identified in bacteria such as *E. coli *[26]. Starting with an understanding of metabolic states in terms of a pooling structure (Figure 1), the present study focused on an in depth *in silico *investigation of the changes that occur in a 'normal' and a genetically 'variant' human erythrocyte. The chief results from the analysis herein were: 1) transport fluxes of bases can be used to characterize changes in the intracellular cofactor pools and dominant metabolic pathways, 2) internal charge ratios can be reflected in cofactor exchange rate response signatures, both of which are affected by genetic variants that have pathological consequences, and 3) the dynamic sequence of interactions that occur within a cell over time can be affected by pathological genetic variants.

Dual perturbation experiments were critical in eliciting the different capabilities among the different genetic variants, thus an external perturbation was necessary. Since the enzymopathies considered involved the redox and energy producing capabilities of the RBC and these are two of its essential metabolic functions, the relevant perturbation conditions are altered redox and energy loads. When a cell changes from one steady state to another, there may be changes in the steady state concentrations, steady state fluxes, and/or the hierarchical interactions that occur over time. We investigated these changes in the red cell under various conditions, including clinically relevant disease states.

### Steady State Analyses

Calculation of the steady state concentrations and fluxes of the nRBC at various redox and energy loads illustrated that the two functional states can be distinguished according to the ADE, ADO, HX, and INO transport fluxes. This observation led to a reduced and experimentally accessible view of the functional metabolic red cell (Figure 1) that maintains information about its functional metabolic state.

An analysis comparing causal SNP variants and the normal red blood cell (Figures 3 and 4) demonstrated that the simplified view of the RBC could distinguish a range of pathophysiological states. The less severe G6PD variants could be differentiated from the more pathological chronic hemolytic patients based on changes in the transport fluxes, as depicted in Figure 3. These results suggested that knowing the state of transporters can directly inform the intracellular metabolic state of the cell. The findings also highlight the importance of applying perturbations to cells (in the wet lab and dry lab alike) in order to understand the functional capabilities and differences between cells that have genetic differences or environmental stresses. The important role of transporters for

### Dynamic Analyses

In order to investigate the dynamic changes of the network under different conditions we calculated the time scales at which different metabolites pool together [22]. The resulting dynamic pooling plots allowed global assessment of the network pooling structure. The responses of the cell to each type of fixed perturbation resulted in different alterations in the resulting pooling structure in the metabolic network, however both types of perturbations resulted in changes in pooling of nucleotide salvage metabolites such as adenosine, adenine, hypoxanthine, inosine, and ribose-1-phosphate. The significance of this result can be appreciated in consideration of the steady state changes (and the resulting distillation of the network shown in Figure 1B) and topological considerations. The two objectives, ATP production and NADPH production, compete for available G6P. Additionally, the nucleotide salvage pathway connects the two by virtue of the importance of ATP/ADP for glycolysis and the ribose phosphates in the non-oxidative branch of the pentose phosphate pathway. Thus although NADPH and ATP generation are linked at the top and bottom of their related pathways in the red cell, the dynamic response to an ATP load however will not be the direct opposite of a load on NADPH because the response times of enzymes in the different pathways differ significantly.

The non-chronic and chronic G6PD variants exhibited marked differences in the pooling of metabolites on progressively slower time scales. In general, the more severe, chronic G6PD variants exhibited a 'disrupted' pooling structure of the network. The complex series of interactions and aggregate pool formation over progressive time scales are difficult to contemplate individually. As highlighted in Figure 6, metabolite pooling typically occured within pathways on faster time scales and between pathways on slower time scales. Analysis of the nRBC and vRBC dynamics under redox loads illustrated that disruptions altering the pooling behavior between metabolites within a pathway, subsequently disrupted interactions between pathways on the slower time scales. For the G6PD variants, this was reflected by changes in the interactions between the pentose phosphate pathway and the nucleotide salvage pathway. Thus, not all G6PD variants had the same metabolic disruptions in their pathways and furthermore, a red cell with G6PD deficiency was not the same as a red cell with a decreased ability to tolerate an oxidative load.

It was observed that even PK variants were able to maintain their energy charge [24] in the 85–90% range [16], however it was not clear if the dynamics of the PK variants under higher energy loads were similar to that of the normal red cell. Investigations of the pooling structure suggested that indeed the dynamics of pool formation in PK variants, in contrast to the G6PD variants, were effectively the same as normal red cells, but with a diminished ability to respond to energy loads.

## Conclusion

Models can be used to simplify what appears complex and in some cases reveal complexity in areas that appear to be simple. Wet lab experiments regularly make use of dual perturbation designs, surprisingly however, such designs have not been employed in dry lab experiments extensively. Herein we have shown that dual perturbations *in silico *are much more informative that single perturbation computational experiments. This was illustrated when the cell was exposed to altered external demands as well as altered rate expression parameters resulting from causal genetic mutations. Analysis of the time scale hierarchy of network dynamics in the context of genetic mutations also revealed a disruption of the normal interactions between metabolites on different time scales. These results suggest that in rough analogy to heterochronic mutants and organogenesis [27-29], the dynamics within metabolic networks may be disrupted in pathological states. Future experiments investigating and testing these hypotheses are worth investigation. It is conceivable that detecting altered pathway dynamics could be an approach for identifying sub-clinical disease or pathological changes in health that are not yet clinically identifiable.

There has been a growing literature concerned with computationally driven experimental design approaches in systems biology [10,30-33]. These wide ranging approaches have been used to address different aspects of biological complexity and have been employed to make predictions which may be used to test hypotheses, engineer microbes, or even build synthetic networks. Here we used a kinetic model of a biochemical network for perturbational analysis of genetic and environmental factors. The use of kinetic models to assist in the characterization and understanding of the genotype-phenotype relationship and disease states is likely to become a more valuable in the future, as models play a more active role in providing predictions and hypotheses to be tested experimentally, particularly by employing dual perturbation designs. Here we suggest that measurements of uptake rates of cofactor precursors and disruption of time scale hierarchy could be used to estimate the internal state cells and how genetic variants differ from a normal (or a reference) cell in response to external perturbations. The predictions put forth can serve as hypotheses to be tested experimentally in future studies.

## Methods

The kinetic model of human red cell metabolism accounting for small metabolite allosteric regulation as originally described by [14,15] and implemented in Mathematica^® ^(Wolfram Research, Chicago, IL) [13] was used for the analyses. Steady state concentrations were calculated using Mathematica's numerical root solving functions and solving the mass balance equations at steady state (error for each equation < 10^-15^) for the set of metabolite concentrations. Steady state fluxes were calculated by substituting the steady state concentrations into the corresponding reaction rate law. The implementation of the model in Mathematica is freely available for download at systemsbiology.ucsd.edu.

### Application of redox and energy loads

Redox loads were applied by increasing the rate constant for the first order load on glutathione (reduced form) (GSHR reaction; a drain on glutathione) that is already part of the model. The maximum coefficient for the normal erythrocyte was 7.5/hr. Most of the variants could not tolerate a load this high, although some of the variants could tolerate an even higher load. The high and low redox loads between the normal cell and the G6PD variants for the comparative study were 2.5 and 0.5 mM/hr, respectively (the maximum applicable load was limited by some of the variants).

Energy loads were applied by adjusting the rate constant for the first order load on ATP (ATPase reaction; a drain on ATP) that is already in the model. The maximum coefficient was 1.6/hr, although many of the variants could not tolerate this high of a load. The high and low energy loads between the normal cell and the PK variants for the comparative study were 0.6 and 0.2 mM/hr, respectively.

### Implementation of kinetic variants

The SNP variants were implemented *in silico *by varying the appropriate kinetic parameters (e.g. V_max_, K_i_, etc) that were measured for individual patients [18-20] in the rate equations for G6PDH and PK, as appropriate. In order to account for any systematic measurement errors between different labs, the values of the patient variants were scaled with respect to the 'normal' control values measured by the particular investigators. This was carried out in the same manner as described previously [16].

### Analysis of dynamics

Decomposition of the dynamics and subsequent characterization of the pooling within the network has been developed and previously described in detail [22] and is described briefly here. Following common practice in dynamical systems analysis, linearization of the mass conservation equations for a chemical reacting system around the steady state yields,

(2)

where J is the nxn Jacobian matrix (n = the number of dynamic concentration variables), and *x'*(= *x *- *x*_ss_) is the deviation vector of the concentration variables from the steady state (*x*_ss_). Temporal decomposition is then carried out, and the modal matrix is constructed by applying a similarity transformation to the Jacobian, J = MΛM^-1^, and redefining the variables as modal variables, m = M^-1^x, so that Equation (2) becomes,

(3)

as described previously [23]. The modal variables, m, are dynamically independent. Aggregate pool formation among metabolites was determined through the calculation of the angles between columns in the modal matrix,

(4)

in which  refers to the magnitude of the *i*^th ^column of the modal matrix and *ϑ*_*ij *_refers to the angle between the *i*^th ^and *j*^th ^columns of the modal matrix.

In order to identify the time scales at which pool formation occurs, we compute the angle between two columns as a function of an index *k *that runs from 1 to *n *time scales. As each row of the modal matrix is removed (*k *increases by one) the angle is recomputed to form a series of angles as a function of *k*; i.e., *ϑ*_*ij *_*(k)*, *k *= 1, 2, ..., n. If the angle *ϑ*_*ij *_*(k) *is close to zero, the two columns are correlated at and above that *k *value and the two corresponding concentrations will move in tandem for the subsequent time scales, thus forming an aggregate variable or a pool.

## Authors' contributions

NJ conceived of the study, carried out the calculations, and drafted the initial draft of the manuscript. NJ and BOP analyzed the results and revised the manuscript.

## Supplementary Material

Additional file 1 **Steady state concentrations and fluxes**. The steady state concentrations of the metabolites and the steady state fluxes of the reactions are provided in the tables. Click here for file

Additional file 2 **Tiled pooled plots of the red cell**. Tiled pooled plots of the normal and variant red cell models under different stress conditions (Figure 1–15). Maps of the qualitative changes in fluxes in response to redox and energy loads (Figure 16). Click here for file
