# Supplementary material for: Using in silico models to simulate dual perturbation experiments: procedure development and interpretation of outcomes
Source: BMC Syst Biol. 2009 Apr 30;3:44. doi: 10.1186/1752-0509-3-44 (PMC2689188; doi:10.1186/1752-0509-3-44)
Supplement: Additional file 2 — Tiled pooled plots of the red cell. Tiled pooled plots of the normal and variant red cell models under different stress conditions (Figure 1–15). Maps of the qualitative changes in fluxes in response to redox and energy loads (Figure 16). [file 1752-0509-3-44-S2.pdf]

	G6P	F6P	FDP	DHAP	GAP	DPG13	DPG23	PG3	PG2	PEP	PYR	LAC	NADH	GL6P	GO6P	NADPH	GSH	RU5P	R5P	X5P	S7P	E4P	ADO	AMP	ADP	ATP	PRPP	IMP	INO	HX	R1P	ADE	NAI	KI
G6P		1	23	22	22	25	27	25	25	25	27	27	27	12	19	31	31	26	26	26	26	20	31	31	31	31	30	29	26	26	26	30	31	0
F6P	4		23	22	22	25	27	25	25	25	27	27	27	12	19	31	31	26	26	26	26	20	31	31	31	31	30	29	26	26	26	30	31	0
FDP	23	23		14	14	25	27	25	25	25	27	27	27	23	26	31	31	26	26	26	26	22	31	31	31	31	30	29	26	26	26	31	31	0
DHAP	23	23	14		3	25	27	25	25	25	27	27	27	22	26	31	31	26	26	26	26	22	31	31	31	31	30	29	26	26	26	31	31	0
GAP	23	23	14	3		25	27	25	25	25	27	27	27	22	26	31	31	26	26	26	26	22	31	31	31	31	30	29	26	26	26	31	31	0
DPG13	24	24	24	24	24		27	22	22	22	27	27	27	25	25	31	31	26	26	26	26	25	24	31	31	31	30	29	26	26	26	30	31	0
DPG23	26	26	27	26	26	26		27	27	27	27	27	27	27	27	31	31	27	27	27	27	27	31	28	28	28	30	29	27	30	27	31	29	0
PG3	24	24	24	24	24	22	26		8	8	27	27	27	25	25	31	31	26	26	26	26	25	31	31	31	31	30	29	26	30	26	31	31	0
PG2	24	24	24	24	24	22	26	8		5	27	27	27	25	25	31	31	26	26	26	26	25	31	31	31	31	30	29	26	30	26	31	31	0
PEP	24	24	24	24	24	22	26	8	5		27	27	27	25	25	31	31	26	26	26	26	25	31	31	31	31	30	29	26	30	26	31	31	0
PYR	29	29	29	29	29	29	26	27	27	27		25	25	27	27	24	24	27	27	27	27	27	31	31	31	31	31	29	27	31	27	31	31	0
LAC	29	29	29	29	29	29	26	27	27	27	24		24	27	27	25	25	27	27	27	27	27	31	31	31	31	31	29	27	31	27	31	31	0
NADH	29	29	29	29	29	29	26	27	27	27	23	23		27	27	25	25	27	27	27	27	27	31	31	31	31	31	29	27	31	27	31	31	0
GL6P	25	25	25	25	25	25	27	25	25	25	29	29	29		19	31	31	19	19	19	26	24	31	31	31	31	30	29	19	19	19	30	31	0
GO6P	25	25	25	25	25	25	27	25	25	25	29	29	29	25		31	31	26	26	26	26	20	31	31	31	31	30	29	26	26	26	30	31	0
NADPH	25	25	25	25	25	27	26	27	27	27	23	24	24	25	25		1	31	31	31	31	31	29	29	30	31	30	31	31	30	31	30	31	0
GSH	25	25	25	25	25	27	26	27	27	27	23	24	24	25	25	1		31	31	31	31	31	29	29	30	31	30	31	31	30	31	30	31	0
RU5P	25	25	25	25	25	25	27	25	25	25	29	29	29	25	20	25	25		7	7	26	26	31	31	31	31	30	29	15	17	7	30	31	0
R5P	25	25	25	25	25	25	27	25	25	25	29	29	29	25	20	25	25	12		13	26	26	31	31	31	31	30	29	15	17	13	30	31	0
X5P	25	25	25	25	25	25	27	25	25	25	29	29	29	25	20	25	25	7	12		26	26	31	31	31	31	30	29	15	17	1	30	31	0
S7P	25	25	25	25	25	25	27	25	25	25	29	24	29	25	25	23	23	20	20	20		26	31	31	31	31	30	30	26	26	26	30	31	0
E4P	25	25	25	25	25	25	27	25	25	25	29	24	29	25	25	20	20	23	22	23	22		31	31	31	31	30	29	26	26	26	30	31	0
ADO	26	26	26	26	26	28	28	28	28	28	29	29	29	26	26	28	28	26	26	26	26	26		10	28	28	30	31	31	30	31	30	31	0
AMP	26	26	26	26	26	27	28	28	28	28	29	29	29	26	26	28	28	26	26	26	26	26	9		27	27	30	31	31	31	31	21	31	0
ADP	26	26	26	26	26	26	28	28	28	28	29	29	29	26	26	28	28	26	26	26	26	26	22	17		27	31	31	31	31	31	31	31	0
ATP	26	26	26	26	26	26	28	28	28	28	29	29	29	26	26	28	28	26	26	26	26	26	24	22	1		31	29	31	31	31	31	29	0
PRPP	26	26	26	26	26	27	28	28	28	28	29	29	29	26	26	28	28	26	26	26	26	26	26	24	1	1		30	30	30	30	27	31	0
IMP	26	26	26	26	26	28	28	28	28	28	29	29	29	26	26	29	29	26	26	26	26	26	22	22	1	1	1		29	30	29	30	29	0
INO	25	25	25	25	25	25	27	25	25	25	29	29	29	25	20	25	25	16	16	16	20	22	26	26	26	26	26	26		17	10	30	31	0
HX	25	25	25	25	25	25	28	25	25	25	29	29	29	25	20	28	28	16	16	16	25	25	26	26	26	26	26	26	16		17	30	31	0
R1P	25	25	25	25	25	25	27	25	25	25	29	29	29	25	20	25	25	15	15	15	20	22	26	26	26	26	26	26	16	16		30	31	0
ADE	27	27	27	27	27	27	28	27	27	27	29	29	29	27	27	29	29	27	27	27	27	27	27	27	27	27	27	27	27	27	27		31	0
NAI	30	30	30	30	30	30	30	30	30	30	30	30	30	30	30	30	30	30	30	30	30	30	30	30	30	30	30	30	30	30	30	30		30
KI	30	30	30	30	30	30	30	30	30	30	30	30	30	30	30	30	30	30	30	30	30	30	30	30	30	30	30	30	30	30	30	30	29	

Supplemental Figure 1

	G6P	F6P	FDP	DHAP	GAP	DPG13	DPG23	PG3	PG2	PEP	PYR	LAC	NADH	GL6P	GO6P	NADPH	GSH	RU5P	R5P	X5P	S7P	E4P	ADO	AMP	ADP	ATP	PRPP	IMP	INO	HX	R1P	ADE	NAI	KI
G6P		1	25	22	22	25	27	25	25	25	27	27	27	13	19	31	31	26	26	26	26	20	31	31	31	31	30	29	26	26	26	31	31	0
F6P	1		25	22	22	25	27	25	25	25	27	27	27	13	19	31	31	26	26	26	26	20	31	31	31	31	30	29	26	26	26	31	31	0
FDP	23	23		14	14	25	27	25	25	25	27	27	27	26	25	31	31	26	26	26	26	23	31	31	31	31	31	29	26	26	26	31	31	0
DHAP	22	22	14		3	25	27	25	25	25	27	27	27	26	26	31	31	26	26	26	26	24	31	31	31	28	31	29	26	26	26	31	31	0
GAP	22	22	14	3		25	27	25	25	25	27	27	27	26	26	31	31	26	26	26	26	24	31	31	31	28	31	29	26	26	26	31	31	0
DPG13	25	25	25	25	25		27	22	22	22	27	27	27	25	25	31	31	26	26	26	26	25	31	31	31	28	30	29	26	30	26	31	31	0
DPG23	27	27	27	27	27	27		27	27	27	27	27	27	27	27	31	31	27	27	27	27	27	28	28	28	28	31	28	27	30	27	31	29	0
PG3	25	25	25	25	25	22	27		8	8	27	27	27	25	25	31	31	26	26	26	27	25	31	31	31	28	31	29	26	30	26	31	31	0
PG2	25	25	25	25	25	22	27	8		5	27	27	27	25	25	31	31	26	26	26	27	25	31	31	31	28	31	29	26	30	26	31	31	0
PEP	25	25	25	25	25	22	27	8	5		27	27	27	25	25	31	31	26	26	26	27	25	31	31	31	28	31	29	26	30	26	31	31	0
PYR	27	27	27	27	27	27	27	27	27	27		25	25	27	27	24	24	27	27	27	27	27	31	31	31	31	31	29	27	31	27	31	31	0
LAC	27	27	27	27	27	27	27	27	27	27	25		24	27	27	25	25	27	27	27	27	27	31	31	31	31	31	29	27	31	27	31	31	0
NADH	27	27	27	27	27	27	27	27	27	27	25	24		27	27	25	25	27	27	27	27	27	31	31	31	31	31	29	27	31	27	31	31	0
GL6P	12	12	23	22	22	25	27	25	25	25	27	27	27		19	31	31	19	19	19	26	24	31	31	31	31	30	29	19	19	19	30	31	0
GO6P	19	19	26	26	26	25	27	25	25	25	27	27	27	19		31	31	26	26	26	26	20	31	31	31	31	30	29	26	26	26	30	31	0
NADPH	31	31	31	31	31	31	31	31	31	31	24	25	25	31	31		1	31	31	31	31	31	29	30	30	31	30	31	31	30	31	28	31	0
GSH	31	31	31	31	31	31	31	31	31	31	24	25	25	31	31	1		31	31	31	31	31	29	30	30	31	30	31	31	30	31	28	31	0
RU5P	26	26	26	26	26	26	27	26	26	26	27	27	27	19	26	31	31		12	7	26	26	31	31	31	31	30	29	15	16	15	30	31	0
R5P	26	26	26	26	26	26	27	26	26	26	27	27	27	19	26	31	31	7		12	26	26	31	31	31	31	30	29	15	16	15	30	31	0
X5P	26	26	26	26	26	26	27	26	26	26	27	27	27	19	26	31	31	7	13		26	26	31	31	31	31	30	29	15	16	15	30	31	0
S7P	26	26	26	26	26	26	27	26	26	26	27	27	27	26	26	31	31	26	26	26		26	31	31	31	31	30	29	26	26	26	30	31	0
E4P	20	20	22	22	22	25	27	25	25	25	27	27	27	24	20	31	31	26	26	26	26		31	31	31	31	30	29	26	26	26	31	31	0
ADO	31	31	31	31	31	24	31	31	31	31	31	31	31	31	31	29	29	31	31	31	31	31		16	27	28	30	31	31	30	31	30	31	0
AMP	31	31	31	31	31	31	28	31	31	31	31	31	31	31	31	29	29	31	31	31	31	31	10		27	27	30	31	31	31	31	30	31	0
ADP	31	31	31	31	31	31	28	31	31	31	31	31	31	31	31	30	30	31	31	31	31	31	28	27		25	31	22	31	31	31	31	31	0
ATP	31	31	31	31	31	31	28	31	31	31	31	31	31	31	31	31	31	31	31	31	31	31	28	27	27		31	27	31	31	31	31	29	0
PRPP	30	30	30	30	30	30	30	30	30	30	31	31	31	30	30	30	30	30	30	30	30	30	30	30	31	31		30	30	30	30	29	31	0
IMP	29	29	29	29	29	29	29	29	29	29	29	29	29	29	29	31	31	29	29	29	30	29	31	31	31	29	30		29	30	29	30	29	0
INO	26	26	26	26	26	26	27	26	26	26	27	27	27	19	26	31	31	15	15	15	26	26	31	31	31	31	30	29		16	10	30	31	0
HX	26	26	26	26	26	26	30	30	30	30	31	31	31	19	26	30	30	17	17	17	26	26	30	31	31	31	30	30	17		16	30	31	0
R1P	26	26	26	26	26	26	27	26	26	26	27	27	27	19	26	31	31	7	13	1	26	26	31	31	31	31	30	29	10	17		30	31	0
ADE	30	30	31	31	31	30	31	31	31	31	31	31	31	30	30	30	30	30	30	30	30	30	30	21	31	31	27	30	30	30	30		31	0
NAI	31	31	31	31	31	31	29	31	31	31	31	31	31	31	31	31	31	31	31	31	31	31	31	31	31	29	31	29	31	31	31	31		30
KI	0	0	0	0	0	0	0	0	0	0	0	0	0	0	0	0	0	0	0	0	0	0	0	0	0	0	0	0	0	0	0	0	30	

Supplemental Figure 2

	G6P	F6P	FDP	DHAP	GAP	DPG13	DPG23	PG3	PG2	PEP	PYR	LAC	NADH	GL6P	GO6P	NADPH	GSH	RU5P	R5P	X5P	S7P	E4P	ADO	AMP	ADP	ATP	PRPP	IMP	INO	HX	R1P	ADE	NAI	KI
G6P		1	22	22	22	25	27	25	25	25	27	27	27	14	19	31	31	26	26	26	26	20	31	31	31	31	30	29	26	26	26	30	31	0
F6P	1		22	22	22	25	27	25	25	25	27	27	27	14	19	31	31	26	26	26	26	20	31	31	31	31	30	29	26	26	26	30	31	0
FDP	23	23		13	13	25	27	25	25	25	27	27	27	23	26	31	31	26	26	26	26	22	31	31	31	31	30	29	26	26	26	30	31	0
DHAP	22	22	14		3	25	27	25	25	25	27	27	27	23	26	31	31	26	26	26	26	22	31	31	31	31	30	29	26	26	26	30	31	0
GAP	22	22	14	3		25	27	25	25	25	27	27	27	23	26	31	31	26	26	26	26	22	31	31	31	31	30	29	26	26	26	30	31	0
DPG13	25	25	25	25	25		27	22	22	22	27	27	27	25	25	31	31	26	26	26	26	25	24	31	31	31	30	29	26	26	26	30	31	0
DPG23	27	27	27	27	27	27		27	27	27	27	27	27	27	27	31	31	27	27	27	27	27	28	28	28	28	30	29	27	30	27	30	29	0
PG3	25	25	25	25	25	22	27		5	8	27	27	27	26	26	31	31	26	26	26	26	25	31	31	31	31	30	29	26	30	26	30	31	0
PG2	25	25	25	25	25	22	27	8		5	27	27	27	26	26	31	31	26	26	26	26	25	31	31	31	31	30	29	26	30	26	30	31	0
PEP	25	25	25	25	25	22	27	8	5		27	27	27	26	26	31	31	26	26	26	26	25	31	31	31	31	30	29	26	30	26	30	31	0
PYR	27	27	27	27	27	27	27	27	27	27		25	25	27	27	24	24	27	27	27	27	27	31	31	31	31	31	29	27	31	27	31	31	0
LAC	27	27	27	27	27	27	27	27	27	27	25		24	27	27	25	25	27	27	27	27	27	31	31	31	31	31	29	27	31	27	31	31	0
NADH	27	27	27	27	27	27	27	27	27	27	25	24		27	27	25	25	27	27	27	27	27	31	31	31	31	31	29	27	31	27	31	31	0
GL6P	12	12	23	22	22	25	27	25	25	25	27	27	27		19	31	31	19	19	19	26	20	31	31	31	31	30	29	19	19	19	30	31	0
GO6P	19	19	26	26	26	25	27	25	25	25	27	27	27	19		31	31	26	26	26	26	20	31	31	31	31	30	29	26	26	26	30	31	0
NADPH	31	31	31	31	31	31	31	31	31	31	24	25	25	31	31		1	31	31	31	31	31	29	29	30	31	30	31	31	30	31	30	31	0
GSH	31	31	31	31	31	31	31	31	31	31	24	25	25	31	31	1		31	31	31	31	31	29	29	30	31	30	31	31	30	31	30	31	0
RU5P	26	26	26	26	26	26	27	26	26	26	27	27	27	19	26	31	31		7	1	26	26	31	31	31	31	30	30	15	16	7	30	31	0
R5P	26	26	26	26	26	26	27	26	26	26	27	27	27	19	26	31	31	7		1	26	26	31	31	31	31	30	30	15	16	12	30	31	0
X5P	26	26	26	26	26	26	27	26	26	26	27	27	27	19	26	31	31	7	13		26	26	31	31	31	31	30	30	15	16	1	30	31	0
S7P	26	26	26	26	26	26	27	26	26	26	27	27	27	26	26	31	31	26	26	26		26	31	31	31	31	30	30	26	26	26	30	31	0
E4P	20	20	22	22	22	25	27	25	25	25	27	27	27	24	20	31	31	26	26	26	26		31	31	31	31	30	29	26	26	26	30	31	0
ADO	31	31	31	31	31	24	31	31	31	31	31	31	31	31	31	29	29	31	31	31	31	31		9	27	28	30	31	31	30	31	30	31	0
AMP	31	31	31	31	31	31	28	31	31	31	31	31	31	31	31	29	29	31	31	31	31	31	10		27	27	30	31	31	31	31	21	31	0
ADP	31	31	31	31	31	31	28	31	31	31	31	31	31	31	31	30	30	31	31	31	31	31	28	27		27	31	31	31	31	31	31	31	0
ATP	31	31	31	31	31	31	28	31	31	31	31	31	31	31	31	31	31	31	31	31	31	31	28	27	27		31	27	31	31	31	31	29	0
PRPP	30	30	30	30	30	30	30	30	30	30	31	31	31	30	30	30	30	30	30	30	30	30	30	30	31	31		30	30	30	30	28	31	0
IMP	29	29	29	29	29	29	29	29	29	29	29	29	29	29	29	31	31	29	29	29	30	29	31	31	31	29	30		29	30	30	30	29	0
INO	26	26	26	26	26	26	27	26	26	26	27	27	27	19	26	31	31	15	15	15	26	26	31	31	31	31	30	29		16	10	30	31	0
HX	26	26	26	26	26	26	30	30	30	30	31	31	31	19	26	30	30	17	17	17	26	26	30	31	31	31	30	30	17		16	30	31	0
R1P	26	26	26	26	26	26	27	26	26	26	27	27	27	19	26	31	31	7	13	1	26	26	31	31	31	31	30	29	10	17		30	31	0
ADE	30	30	31	31	31	30	31	31	31	31	31	31	31	30	30	30	30	30	30	30	30	30	30	21	31	31	27	30	30	30	30		31	0
NAI	31	31	31	31	31	31	29	31	31	31	31	31	31	31	31	31	31	31	31	31	31	31	31	31	31	29	31	29	31	31	31	31		30
KI	0	0	0	0	0	0	0	0	0	0	0	0	0	0	0	0	0	0	0	0	0	0	0	0	0	0	0	0	0	0	0	0	30	

Supplemental Figure 3

	G6P	F6P	FDP	DHAP	GAP	DPG13	DPG23	PG3	PG2	PEP	PYR	LAC	NADH	GL6P	GO6P	NADPH	GSH	RU5P	R5P	X5P	S7P	E4P	ADO	AMP	ADP	ATP	PRPP	IMP	INO	HX	R1P	ADE	NAI	KI
G6P		1	22	22	22	24	26	24	24	24	26	26	26	22	22	26	26	24	24	24	25	24	29	29	29	29	29	29	24	24	24	29	30	30
F6P	1		22	22	22	24	26	24	24	24	26	26	26	22	22	26	26	24	24	24	25	24	29	29	29	29	29	29	24	24	24	29	30	30
FDP	23	23		14	14	24	26	24	24	24	26	26	26	21	14	26	26	21	21	21	25	23	29	29	29	29	29	29	21	21	21	29	30	30
DHAP	22	22	14		3	24	26	24	24	24	26	26	26	19	13	26	26	21	21	21	25	23	29	29	29	29	29	29	21	21	21	29	30	30
GAP	22	22	14	3		24	26	24	24	24	26	26	26	19	13	26	26	21	21	21	25	23	29	29	29	29	29	29	21	21	21	29	30	30
DPG13	25	25	25	25	25		26	22	22	22	26	26	26	24	24	26	26	24	24	24	25	24	29	29	29	29	29	29	24	24	24	29	30	30
DPG23	27	27	27	27	27	27		26	26	26	26	26	26	26	26	29	29	26	26	26	26	26	28	28	28	28	29	29	26	29	26	29	30	30
PG3	25	25	25	25	25	22	27		8	8	26	26	26	24	24	29	29	24	24	24	25	24	29	29	29	29	29	29	24	24	24	29	30	30
PG2	25	25	25	25	25	22	27	8		5	26	26	26	24	24	29	29	24	24	24	25	24	29	29	29	29	29	29	24	24	24	29	30	30
PEP	25	25	25	25	25	22	27	8	5		26	26	26	24	24	29	29	24	24	24	25	24	29	29	29	29	29	29	24	24	24	29	30	30
PYR	27	27	27	27	27	27	27	27	27	27		23	23	26	26	29	29	26	26	26	26	26	29	29	29	29	29	29	26	29	26	29	30	30
LAC	27	27	27	27	27	27	27	27	27	27	25		23	26	26	29	29	26	26	26	26	26	29	29	29	29	29	29	26	29	26	29	30	30
NADH	27	27	27	27	27	27	27	27	27	27	25	24		26	26	29	29	26	26	26	26	26	29	29	29	29	29	29	26	29	26	29	30	30
GL6P	12	12	23	22	22	25	27	25	25	25	27	27	27		18	29	29	19	21	19	25	24	29	29	29	29	29	29	19	19	19	29	30	30
GO6P	19	19	26	26	26	25	27	25	25	25	27	27	27	19		29	29	25	25	25	25	25	29	29	29	29	29	29	25	25	25	29	30	30
NADPH	31	31	31	31	31	31	31	31	31	31	24	25	25	31	31		1	29	29	29	29	26	26	26	26	25	26	29	29	29	29	26	30	30
GSH	31	31	31	31	31	31	31	31	31	31	24	25	25	31	31	1		29	29	29	29	26	26	26	26	25	26	29	29	29	29	26	30	30
RU5P	26	26	26	26	26	26	27	26	26	26	27	27	27	19	26	31	31		1	1	7	21	29	29	29	29	29	29	15	16	1	29	30	30
R5P	26	26	26	26	26	26	27	26	26	26	27	27	27	19	26	31	31	7		1	13	21	29	29	29	29	29	29	15	16	1	29	30	30
X5P	26	26	26	26	26	26	27	26	26	26	27	27	27	19	26	31	31	7	13		1	21	29	29	29	29	29	29	15	16	1	29	30	30
S7P	26	26	26	26	26	26	27	26	26	26	27	27	27	26	26	31	31	26	26	26		19	29	29	29	29	29	29	16	19	15	29	30	30
E4P	20	20	22	22	22	25	27	25	25	25	27	27	27	24	20	31	31	26	26	26	26		29	29	29	29	29	29	21	21	21	29	30	30
ADO	31	31	31	31	31	24	31	31	31	31	31	31	31	31	31	29	29	31	31	31	31	31		9	26	26	26	29	29	29	29	24	30	30
AMP	31	31	31	31	31	31	28	31	31	31	31	31	31	31	31	29	29	31	31	31	31	31	10		22	26	26	29	29	29	29	21	30	30
ADP	31	31	31	31	31	31	28	31	31	31	31	31	31	31	31	30	30	31	31	31	31	31	28	27		22	17	29	29	29	29	21	30	30
ATP	31	31	31	31	31	31	28	31	31	31	31	31	31	31	31	31	31	31	31	31	31	31	28	27	27		26	29	29	29	29	26	30	30
PRPP	30	30	30	30	30	30	30	30	30	30	31	31	31	30	30	30	30	30	30	30	30	30	30	30	31	31		28	29	28	29	21	30	30
IMP	29	29	29	29	29	29	29	29	29	29	29	29	29	29	29	31	31	29	29	29	30	29	31	31	31	29	30		29	28	29	29	28	30
INO	26	26	26	26	26	26	27	26	26	26	27	27	27	19	26	31	31	15	15	15	26	26	31	31	31	31	30	29		16	11	29	30	30
HX	26	26	26	26	26	26	30	30	30	30	31	31	31	19	26	30	30	17	17	17	26	26	30	31	31	31	30	30	17		16	29	30	30
R1P	26	26	26	26	26	26	27	26	26	26	27	27	27	19	26	31	31	7	13	1	26	26	31	31	31	31	30	29	10	17		29	30	30
ADE	30	30	31	31	31	30	31	31	31	31	31	31	31	30	30	30	30	30	30	30	30	30	30	21	31	31	27	30	30	30	30		30	30
NAI	31	31	31	31	31	31	29	31	31	31	31	31	31	31	31	31	31	31	31	31	31	31	31	31	31	29	31	29	31	31	31	31		29
KI	0	0	0	0	0	0	0	0	0	0	0	0	0	0	0	0	0	0	0	0	0	0	0	0	0	0	0	0	0	0	0	0	30	

Supplemental Figure 4

	G6P	F6P	FDP	DHAP	GAP	DPG13	DPG23	PG3	PG2	PEP	PYR	LAC	NADH	GL6P	GO6P	NADPH	GSH	RU5P	R5P	X5P	S7P	E4P	ADO	AMP	ADP	ATP	PRPP	IMP	INO	HX	R1P	ADE	NAI	KI
G6P		1	22	12	12	24	26	24	24	24	26	26	26	20	22	22	22	22	22	22	23	22	25	25	25	25	28	26	22	22	22	26	29	29
F6P	1		22	12	12	24	26	24	24	24	26	26	26	20	22	22	22	22	22	22	23	22	25	25	25	25	28	26	22	22	22	26	29	29
FDP	23	23		12	12	24	26	24	24	24	26	26	26	18	12	14	14	12	12	12	14	14	25	25	25	25	28	26	14	16	12	26	29	29
DHAP	22	22	14		1	24	26	24	24	24	26	26	26	18	11	1	1	1	1	1	22	12	25	25	25	25	28	26	10	16	1	26	29	29
GAP	22	22	14	3		24	26	24	24	24	26	26	26	18	11	3	3	3	3	3	22	12	25	25	25	25	28	26	10	16	3	26	29	29
DPG13	25	25	25	25	25		25	22	22	22	26	26	26	24	24	23	23	24	24	24	23	23	26	26	26	26	28	26	24	24	24	26	29	29
DPG23	27	27	27	27	27	27		25	25	25	25	25	25	26	26	26	26	26	26	26	26	26	26	26	26	26	28	1	26	25	26	26	29	29
PG3	25	25	25	25	25	22	27		5	8	26	26	26	24	24	24	24	24	24	24	24	24	26	26	26	26	28	26	24	24	24	26	29	29
PG2	25	25	25	25	25	22	27	8		5	26	26	26	24	24	24	24	24	24	24	24	24	26	26	26	26	28	26	24	24	24	26	29	29
PEP	25	25	25	25	25	22	27	8	5		26	26	26	24	24	24	24	24	24	24	24	24	26	26	26	26	28	26	24	24	24	26	29	29
PYR	27	27	27	27	27	27	27	27	27	27		23	23	26	26	24	24	26	26	26	26	26	26	26	26	26	28	25	26	25	26	28	29	29
LAC	27	27	27	27	27	27	27	27	27	27	25		22	26	26	24	24	26	26	26	26	26	26	26	26	26	28	25	26	25	26	28	29	29
NADH	27	27	27	27	27	27	27	27	27	27	25	24		26	26	24	24	26	26	26	26	26	26	26	26	26	28	25	26	25	26	28	29	29
GL6P	12	12	23	22	22	25	27	25	25	25	27	27	27		11	18	18	7	13	13	22	20	25	25	25	25	28	26	16	18	15	26	29	29
GO6P	19	19	26	26	26	25	27	25	25	25	27	27	27	19		11	11	11	11	11	11	19	25	25	25	25	28	26	11	16	11	26	29	29
NADPH	31	31	31	31	31	31	31	31	31	31	24	25	25	31	31		1	1	1	1	1	1	25	25	25	25	28	26	10	16	1	25	29	29
GSH	31	31	31	31	31	31	31	31	31	31	24	25	25	31	31	1		1	1	1	1	1	25	25	25	25	28	26	10	16	1	25	29	29
RU5P	26	26	26	26	26	26	27	26	26	26	27	27	27	19	26	31	31		1	1	1	7	25	25	25	25	28	26	1	16	1	26	29	29
R5P	26	26	26	26	26	26	27	26	26	26	27	27	27	19	26	31	31	7		1	1	1	25	25	25	25	28	26	1	16	1	26	29	29
X5P	26	26	26	26	26	26	27	26	26	26	27	27	27	19	26	31	31	7	13		1	1	25	25	25	25	28	26	1	16	1	26	29	29
S7P	26	26	26	26	26	26	27	26	26	26	27	27	27	26	26	31	31	26	26	26		19	25	25	25	25	28	26	10	16	1	26	29	29
E4P	20	20	22	22	22	25	27	25	25	25	27	27	27	24	20	31	31	26	26	26	26		25	25	25	25	28	26	16	19	15	26	29	29
ADO	31	31	31	31	31	24	31	31	31	31	31	31	31	31	31	29	29	31	31	31	31	31		1	25	25	28	26	25	28	25	21	29	29
AMP	31	31	31	31	31	31	28	31	31	31	31	31	31	31	31	29	29	31	31	31	31	31	10		17	25	28	26	25	29	25	21	29	29
ADP	31	31	31	31	31	31	28	31	31	31	31	31	31	31	31	30	30	31	31	31	31	31	28	27		1	29	26	25	29	25	21	29	29
ATP	31	31	31	31	31	31	28	31	31	31	31	31	31	31	31	31	31	31	31	31	31	31	28	27	27		29	26	25	29	25	25	29	29
PRPP	30	30	30	30	30	30	30	30	30	30	31	31	31	30	30	30	30	30	30	30	30	30	30	30	31	31		28	28	28	28	28	29	29
IMP	29	29	29	29	29	29	29	29	29	29	29	29	29	29	29	31	31	29	29	29	30	29	31	31	31	29	30		26	25	26	26	29	29
INO	26	26	26	26	26	26	27	26	26	26	27	27	27	19	26	31	31	15	15	15	26	26	31	31	31	31	30	29		16	1	26	29	29
HX	26	26	26	26	26	26	30	30	30	30	31	31	31	19	26	30	30	17	17	17	26	26	30	31	31	31	30	30	17		16	28	29	29
R1P	26	26	26	26	26	26	27	26	26	26	27	27	27	19	26	31	31	7	13	1	26	26	31	31	31	31	30	29	10	17		26	29	29
ADE	30	30	31	31	31	30	31	31	31	31	31	31	31	30	30	30	30	30	30	30	30	30	30	21	31	31	27	30	30	30	30		29	29
NAI	31	31	31	31	31	31	29	31	31	31	31	31	31	31	31	31	31	31	31	31	31	31	31	31	31	29	31	29	31	31	31	31		28
KI	0	0	0	0	0	0	0	0	0	0	0	0	0	0	0	0	0	0	0	0	0	0	0	0	0	0	0	0	0	0	0	0	30	

Supplemental Figure 5

	G6P	F6P	FDP	DHAP	GAP	DPG13	DPG23	PG3	PG2	PEP	PYR	LAC	NADH	GL6P	GO6P	NADPH	GSH	RU5P	R5P	X5P	S7P	E4P	ADO	AMP	ADP	ATP	PRPP	IMP	INO	HX	R1P	ADE	NAI	KI
G6P		1	24	24	24	24	26	24	24	24	29	29	29	23	23	27	27	25	25	25	25	25	26	26	26	26	26	26	25	25	25	26	29	30
F6P	1		24	24	24	24	26	24	24	24	29	29	29	23	23	27	27	25	25	25	25	25	26	26	26	26	26	26	25	25	25	26	29	30
FDP	23	23		13	13	24	26	24	24	24	29	29	29	22	22	27	27	25	25	25	25	25	26	26	26	26	26	26	25	25	25	26	29	30
DHAP	22	22	14		3	24	26	24	24	24	29	29	29	22	22	27	27	25	25	25	25	25	26	26	26	26	26	26	25	25	25	26	29	30
GAP	22	22	14	3		24	26	24	24	24	29	29	29	22	22	27	27	25	25	25	25	25	26	26	26	26	26	26	25	25	25	26	29	30
DPG13	25	25	25	25	25		26	22	22	22	29	29	29	24	24	27	27	25	25	25	25	25	26	26	26	26	26	26	25	24	25	26	29	30
DPG23	27	27	27	27	27	27		26	26	26	29	29	29	26	26	27	27	26	26	26	26	26	28	28	28	28	27	28	26	26	26	27	29	30
PG3	25	25	25	25	25	22	27		8	8	29	29	29	24	24	27	27	25	25	25	25	25	26	26	26	26	26	26	25	24	25	26	29	30
PG2	25	25	25	25	25	22	27	8		5	29	29	29	24	24	27	27	25	25	25	25	25	26	26	26	26	26	26	25	24	25	26	29	30
PEP	25	25	25	25	25	22	27	8	5		29	29	29	24	24	27	27	25	25	25	25	25	26	26	26	26	26	26	25	24	25	26	29	30
PYR	27	27	27	27	27	27	27	27	27	27		23	23	29	29	29	29	29	29	29	29	29	28	28	28	28	29	29	29	28	29	29	29	30
LAC	27	27	27	27	27	27	27	27	27	27	25		23	29	29	29	29	29	29	29	29	29	28	28	28	28	29	29	29	28	29	29	29	30
NADH	27	27	27	27	27	27	27	27	27	27	25	24		29	29	29	29	29	29	29	29	29	28	28	28	28	29	29	29	28	29	29	29	30
GL6P	12	12	23	22	22	25	27	25	25	25	27	27	27		18	27	27	25	25	25	25	25	26	26	26	26	26	26	25	24	25	26	29	30
GO6P	19	19	26	26	26	25	27	25	25	25	27	27	27	19		27	27	25	25	25	25	25	26	26	26	26	26	26	25	25	25	26	29	30
NADPH	31	31	31	31	31	31	31	31	31	31	24	25	25	31	31		1	27	27	27	27	27	27	27	27	27	27	27	27	27	27	27	29	30
GSH	31	31	31	31	31	31	31	31	31	31	24	25	25	31	31	1		27	27	27	27	27	27	27	27	27	27	27	27	27	27	27	29	30
RU5P	26	26	26	26	26	26	27	26	26	26	27	27	27	19	26	31	31		12	7	22	23	26	26	26	26	26	26	16	16	15	26	29	30
R5P	26	26	26	26	26	26	27	26	26	26	27	27	27	19	26	31	31	7		12	22	23	26	26	26	26	26	26	16	16	15	26	29	30
X5P	26	26	26	26	26	26	27	26	26	26	27	27	27	19	26	31	31	7	13		22	23	26	26	26	26	26	26	16	16	15	26	29	30
S7P	26	26	26	26	26	26	27	26	26	26	27	27	27	26	26	31	31	26	26	26		20	26	26	26	26	26	26	22	25	22	26	29	30
E4P	20	20	22	22	22	25	27	25	25	25	27	27	27	24	20	31	31	26	26	26	26		26	26	26	26	26	26	23	24	23	26	29	30
ADO	31	31	31	31	31	24	31	31	31	31	31	31	31	31	31	29	29	31	31	31	31	31		9	22	22	24	22	26	26	26	27	29	30
AMP	31	31	31	31	31	31	28	31	31	31	31	31	31	31	31	29	29	31	31	31	31	31	10		1	22	22	1	26	26	26	27	29	30
ADP	31	31	31	31	31	31	28	31	31	31	31	31	31	31	31	30	30	31	31	31	31	31	28	27		1	1	1	26	26	26	27	29	30
ATP	31	31	31	31	31	31	28	31	31	31	31	31	31	31	31	31	31	31	31	31	31	31	28	27	27		1	1	26	25	26	27	29	30
PRPP	30	30	30	30	30	30	30	30	30	30	31	31	31	30	30	30	30	30	30	30	30	30	30	30	31	31		27	26	20	26	21	29	30
IMP	29	29	29	29	29	29	29	29	29	29	29	29	29	29	29	31	31	29	29	29	30	29	31	31	31	29	30		26	27	26	27	29	30
INO	26	26	26	26	26	26	27	26	26	26	27	27	27	19	26	31	31	15	15	15	26	26	31	31	31	31	30	29		16	10	26	29	30
HX	26	26	26	26	26	26	30	30	30	30	31	31	31	19	26	30	30	17	17	17	26	26	30	31	31	31	30	30	17		16	22	29	30
R1P	26	26	26	26	26	26	27	26	26	26	27	27	27	19	26	31	31	7	13	1	26	26	31	31	31	31	30	29	10	17		26	29	30
ADE	30	30	31	31	31	30	31	31	31	31	31	31	31	30	30	30	30	30	30	30	30	30	30	21	31	31	27	30	30	30	30		29	30
NAI	31	31	31	31	31	31	29	31	31	31	31	31	31	31	31	31	31	31	31	31	31	31	31	31	31	29	31	29	31	31	31	31		30
KI	0	0	0	0	0	0	0	0	0	0	0	0	0	0	0	0	0	0	0	0	0	0	0	0	0	0	0	0	0	0	0	0	30	

Supplemental Figure 6

	G6P	F6P	FDP	DHAP	GAP	DPG13	DPG23	PG3	PG2	PEP	PYR	LAC	NADH	GL6P	GO6P	NADPH	GSH	RU5P	R5P	X5P	S7P	E4P	ADO	AMP	ADP	ATP	PRPP	IMP	INO	HX	R1P	ADE	NAI	KI
G6P		1	22	12	12	24	28	24	24	24	24	24	24	19	10	25	25	1	1	1	22	22	26	26	26	26	26	26	15	16	1	27	30	30
F6P	1		22	12	12	24	28	24	24	24	24	24	24	19	10	25	25	4	1	1	22	22	26	26	26	26	26	26	15	16	4	27	30	30
FDP	23	23		12	12	14	28	24	24	24	24	24	24	19	12	25	25	1	1	1	1	12	26	26	26	26	26	26	14	16	12	27	30	30
DHAP	22	22	14		1	17	28	24	24	24	24	24	24	19	10	25	25	1	1	1	1	1	26	26	26	26	26	26	11	16	1	27	30	30
GAP	22	22	14	3		17	28	24	24	24	24	24	24	19	10	25	25	3	1	1	1	3	26	26	26	26	26	26	11	16	3	27	30	30
DPG13	25	25	25	25	25		26	22	22	22	24	24	24	23	23	25	25	23	23	23	23	19	26	26	26	26	26	26	23	23	23	27	30	30
DPG23	27	27	27	27	27	27		26	26	26	26	26	26	28	28	27	27	28	28	28	28	28	28	28	28	28	28	28	28	28	28	28	30	30
PG3	25	25	25	25	25	22	27		8	8	24	24	24	24	24	25	25	24	24	24	24	23	28	28	28	28	26	28	24	24	24	27	30	30
PG2	25	25	25	25	25	22	27	8		5	24	24	24	24	24	25	25	24	24	24	24	23	28	28	28	28	26	28	24	24	24	27	30	30
PEP	25	25	25	25	25	22	27	8	5		24	24	24	24	24	25	25	24	24	24	24	23	28	28	28	28	26	28	24	24	24	27	30	30
PYR	27	27	27	27	27	27	27	27	27	27		24	24	24	24	25	25	24	24	24	24	24	28	28	28	28	28	28	24	24	24	28	30	30
LAC	27	27	27	27	27	27	27	27	27	27	25		23	24	24	25	25	24	24	24	24	24	28	28	28	28	28	28	24	24	24	28	30	30
NADH	27	27	27	27	27	27	27	27	27	27	25	24		24	24	25	25	24	24	24	24	24	28	28	28	28	28	28	24	24	24	28	30	30
GL6P	12	12	23	22	22	25	27	25	25	25	27	27	27		18	25	25	19	19	19	19	22	26	26	26	26	26	26	19	19	19	27	30	30
GO6P	19	19	26	26	26	25	27	25	25	25	27	27	27	19		25	25	10	10	10	10	22	26	26	26	26	26	26	11	16	10	27	30	30
NADPH	31	31	31	31	31	31	31	31	31	31	24	25	25	31	31		1	25	25	25	25	25	29	29	29	29	29	29	25	25	25	29	30	30
GSH	31	31	31	31	31	31	31	31	31	31	24	25	25	31	31	1		25	25	25	25	25	29	29	29	29	29	29	25	25	25	29	30	30
RU5P	26	26	26	26	26	26	27	26	26	26	27	27	27	19	26	31	31		1	1	1	7	26	26	26	26	26	26	7	16	1	27	30	30
R5P	26	26	26	26	26	26	27	26	26	26	27	27	27	19	26	31	31	7		1	1	1	26	26	26	26	26	26	11	16	1	27	30	30
X5P	26	26	26	26	26	26	27	26	26	26	27	27	27	19	26	31	31	7	13		1	1	26	26	26	26	26	26	1	16	1	27	30	30
S7P	26	26	26	26	26	26	27	26	26	26	27	27	27	26	26	31	31	26	26	26		1	26	26	26	26	26	26	11	16	1	27	30	30
E4P	20	20	22	22	22	25	27	25	25	25	27	27	27	24	20	31	31	26	26	26	26		26	26	26	26	26	26	16	16	15	27	30	30
ADO	31	31	31	31	31	24	31	31	31	31	31	31	31	31	31	29	29	31	31	31	31	31		9	26	26	26	25	26	26	26	27	30	30
AMP	31	31	31	31	31	31	28	31	31	31	31	31	31	31	31	29	29	31	31	31	31	31	10		25	26	26	22	26	26	26	27	30	30
ADP	31	31	31	31	31	31	28	31	31	31	31	31	31	31	31	30	30	31	31	31	31	31	28	27		25	1	1	26	26	26	27	30	30
ATP	31	31	31	31	31	31	28	31	31	31	31	31	31	31	31	31	31	31	31	31	31	31	28	27	27		1	26	26	26	26	27	30	30
PRPP	30	30	30	30	30	30	30	30	30	30	31	31	31	30	30	30	30	30	30	30	30	30	30	30	31	31		26	26	26	26	26	30	30
IMP	29	29	29	29	29	29	29	29	29	29	29	29	29	29	29	31	31	29	29	29	30	29	31	31	31	29	30		26	26	26	27	30	30
INO	26	26	26	26	26	26	27	26	26	26	27	27	27	19	26	31	31	15	15	15	26	26	31	31	31	31	30	29		16	1	27	30	30
HX	26	26	26	26	26	26	30	30	30	30	31	31	31	19	26	30	30	17	17	17	26	26	30	31	31	31	30	30	17		16	26	30	30
R1P	26	26	26	26	26	26	27	26	26	26	27	27	27	19	26	31	31	7	13	1	26	26	31	31	31	31	30	29	10	17		27	30	30
ADE	30	30	31	31	31	30	31	31	31	31	31	31	31	30	30	30	30	30	30	30	30	30	30	21	31	31	27	30	30	30	30		30	30
NAI	31	31	31	31	31	31	29	31	31	31	31	31	31	31	31	31	31	31	31	31	31	31	31	31	31	29	31	29	31	31	31	31		30
KI	0	0	0	0	0	0	0	0	0	0	0	0	0	0	0	0	0	0	0	0	0	0	0	0	0	0	0	0	0	0	0	0	30	

Supplemental Figure 7

	G6P	F6P	FDP	DHAP	GAP	DPG13	DPG23	PG3	PG2	PEP	PYR	LAC	NADH	GL6P	GO6P	NADPH	GSH	RU5P	R5P	X5P	S7P	E4P	ADO	AMP	ADP	ATP	PRPP	IMP	INO	HX	R1P	ADE	NAI	KI
G6P		1	20	20	20	25	27	25	25	25	27	27	27	23	24	25	25	23	23	23	23	23	28	28	28	28	28	29	23	29	23	27	29	30
F6P	4		20	20	20	25	27	25	25	25	27	27	27	23	24	25	25	23	23	23	23	23	28	28	28	28	28	29	23	29	23	27	29	30
FDP	24	24		14	14	25	27	25	25	25	27	27	27	24	25	25	25	23	23	23	24	23	28	28	28	28	28	29	23	29	23	27	29	30
DHAP	24	24	14		3	25	27	25	25	25	27	27	27	24	25	25	25	23	23	23	25	23	28	28	28	28	28	29	23	29	23	27	29	30
GAP	24	24	14	3		25	27	25	25	25	27	27	27	24	25	25	25	23	23	23	25	23	28	28	28	28	28	29	23	29	23	27	29	30
DPG13	24	24	24	24	24		27	20	20	20	27	27	27	25	25	25	25	25	25	25	25	25	28	28	28	28	28	29	25	29	25	27	29	30
DPG23	28	28	29	28	28	27		26	26	26	26	26	26	27	27	26	26	27	27	27	27	27	28	28	28	28	28	29	27	29	27	27	29	30
PG3	27	27	27	27	27	15	27		8	8	27	27	27	25	25	25	25	25	25	25	25	25	28	28	28	28	28	29	25	29	25	27	29	30
PG2	27	27	27	27	27	15	27	8		5	27	27	27	25	25	25	25	25	25	25	25	25	28	28	28	28	28	29	25	29	25	27	29	30
PEP	27	27	27	27	27	15	27	8	5		27	27	27	25	25	25	25	25	25	25	25	25	28	28	28	28	28	29	25	29	25	27	29	30
PYR	31	31	31	31	31	27	26	27	27	27		24	24	27	27	24	24	27	27	27	27	27	29	29	29	29	29	29	27	29	27	27	30	30
LAC	31	31	31	31	31	23	26	23	23	23	23		24	27	27	23	23	27	27	27	27	27	29	29	29	29	29	29	27	29	27	27	30	30
NADH	31	31	31	31	31	23	26	23	23	23	23	22		27	27	24	24	27	27	27	27	27	29	29	29	29	29	29	27	29	27	27	30	30
GL6P	26	26	26	26	26	26	27	27	27	27	31	31	31		24	25	25	20	20	20	23	23	28	28	28	28	28	29	20	29	20	27	29	30
GO6P	26	26	26	26	26	26	27	27	27	27	31	31	31	26		25	25	24	24	24	24	25	28	28	28	28	28	29	24	29	24	27	29	30
NADPH	22	22	26	26	26	26	32	32	32	32	31	31	31	26	26		1	25	25	25	25	25	28	28	28	28	29	29	25	29	25	27	30	30
GSH	22	22	26	26	26	26	32	32	32	32	31	31	31	26	26	1		25	25	25	25	25	28	28	28	28	29	29	25	29	25	27	30	30
RU5P	25	25	25	25	25	25	29	27	27	27	30	30	30	26	26	26	26		12	7	23	23	28	28	28	28	28	29	16	29	15	27	29	30
R5P	25	25	25	25	25	25	29	27	27	27	30	30	30	26	26	26	26	12		12	23	23	28	28	28	28	28	29	16	29	15	27	29	30
X5P	25	25	25	25	25	25	29	27	27	27	30	30	30	26	26	26	26	7	12		23	23	28	28	28	28	28	29	16	29	15	27	29	30
S7P	25	25	25	25	25	25	29	27	27	27	30	30	30	26	26	26	26	24	24	24		23	28	28	28	28	28	29	23	29	23	27	29	30
E4P	25	25	19	19	19	25	29	27	27	27	30	30	30	26	26	21	21	24	24	24	24		28	28	28	28	28	29	23	29	23	27	29	30
ADO	29	29	15	10	9	22	29	29	29	29	32	32	32	32	32	32	32	32	32	32	32	21		9	26	27	27	29	28	29	28	28	29	30
AMP	29	29	15	15	15	22	29	29	29	29	32	32	32	32	32	32	32	32	32	32	32	32	9		26	26	27	29	28	29	28	28	29	30
ADP	29	29	29	29	29	29	29	29	29	29	32	32	32	32	32	32	32	32	32	32	32	32	27	15		25	27	29	28	29	28	28	29	30
ATP	29	29	29	29	29	29	29	29	29	29	32	32	32	29	29	32	32	32	32	32	32	32	27	27	24		27	29	28	29	28	28	29	30
PRPP	31	31	32	32	32	32	32	32	32	32	31	31	31	31	31	31	31	31	31	31	31	31	32	32	32	32		27	28	29	28	23	29	30
IMP	28	28	28	28	28	28	28	28	28	28	28	28	28	28	28	31	31	28	28	28	28	28	32	32	29	29	31		29	28	29	28	29	30
INO	25	25	25	25	25	25	29	27	27	27	30	30	30	26	26	26	26	17	17	17	24	23	32	32	32	32	31	28		29	16	27	29	30
HX	25	25	25	25	25	31	32	31	31	31	30	30	30	26	26	31	31	17	17	17	25	25	32	32	32	32	31	31	17		29	29	30	30
R1P	25	25	25	25	25	25	29	27	27	27	30	30	30	26	26	26	26	16	16	16	24	23	32	32	32	32	31	28	17	17		27	29	30
ADE	32	32	32	32	32	32	32	32	32	32	31	31	31	31	31	29	29	31	31	31	31	31	20	20	32	32	31	31	31	31	31		29	30
NAI	32	32	32	32	32	32	32	32	32	32	32	32	32	32	32	32	32	32	32	32	32	32	32	32	32	32	32	32	32	32	32	32		30
KI	0	0	0	0	0	0	0	0	0	0	0	0	0	0	0	0	0	0	0	0	0	0	0	0	0	0	0	0	0	0	0	0	30	

Supplemental Figure 8

	G6P	F6P	FDP	DHAP	GAP	DPG13	DPG23	PG3	PG2	PEP	PYR	LAC	NADH	GL6P	GO6P	NADPH	GSH	RU5P	R5P	X5P	S7P	E4P	ADO	AMP	ADP	ATP	PRPP	IMP	INO	HX	R1P	ADE	NAI	KI
G6P		4	24	24	24	24	27	27	27	27	31	31	31	26	26	26	26	25	25	25	25	25	29	29	29	29	31	28	25	25	25	32	32	0
F6P	1		24	24	24	24	27	27	27	27	31	31	31	26	26	26	26	25	25	25	25	25	29	29	29	29	31	28	25	25	25	32	32	0
FDP	20	20		14	14	24	27	27	27	27	31	31	31	26	26	26	26	25	25	25	25	25	16	16	29	29	32	28	25	25	25	32	32	0
DHAP	20	20	14		3	24	27	27	27	27	31	31	31	26	26	26	26	25	25	25	25	25	11	16	29	29	32	28	25	25	25	32	32	0
GAP	20	20	14	3		24	27	27	27	27	31	31	31	26	26	26	26	25	25	25	25	25	11	16	29	29	32	28	25	25	25	32	32	0
DPG13	25	25	25	25	25		27	22	22	22	27	23	23	26	26	26	26	25	25	25	25	25	22	29	29	29	32	28	25	31	25	32	32	0
DPG23	27	27	27	27	27	27		27	27	27	26	26	26	27	27	32	32	27	27	27	27	27	29	29	29	29	32	28	27	32	27	32	32	0
PG3	25	25	25	25	25	20	26		8	8	27	22	23	27	27	32	32	27	27	27	27	27	29	29	29	29	32	28	27	31	27	32	32	0
PG2	25	25	25	25	25	20	26	8		5	27	22	23	27	27	32	32	27	27	27	27	27	29	29	29	29	32	28	27	31	27	32	32	0
PEP	25	25	25	25	25	20	26	8	5		27	22	23	27	27	32	32	27	27	27	27	27	29	29	29	29	32	28	27	31	27	32	32	0
PYR	27	27	27	27	27	27	26	27	27	27		23	23	31	31	31	31	30	30	30	30	30	32	32	32	32	31	28	30	30	30	31	32	0
LAC	27	27	27	27	27	27	26	27	27	27	24		22	31	31	31	31	30	30	30	30	30	32	32	32	32	31	28	30	30	30	31	32	0
NADH	27	27	27	27	27	27	26	27	27	27	24	24		31	31	31	31	30	30	30	30	30	32	32	32	32	31	28	30	30	30	31	32	0
GL6P	23	23	24	24	24	25	27	25	25	25	27	27	27		26	26	26	26	26	26	26	26	32	32	32	32	31	28	26	26	26	31	32	0
GO6P	24	24	25	25	25	25	27	25	25	25	27	27	27	24		26	26	26	26	26	26	26	32	32	32	32	31	28	26	26	26	31	32	0
NADPH	25	25	25	25	25	25	26	25	25	25	24	23	24	25	25		1	26	26	26	26	21	32	32	32	32	31	31	26	31	26	29	32	0
GSH	25	25	25	25	25	25	26	25	25	25	24	23	24	25	25	1		26	26	26	26	21	32	32	32	32	31	31	26	31	26	29	32	0
RU5P	23	23	23	23	23	25	27	25	25	25	27	27	27	20	24	25	25		12	7	24	24	32	32	32	32	31	28	17	17	15	31	32	0
R5P	23	23	23	23	23	25	27	25	25	25	27	27	27	20	24	25	25	12		12	24	24	32	32	32	32	31	28	17	17	15	31	32	0
X5P	23	23	23	23	23	25	27	25	25	25	27	27	27	20	24	25	25	7	12		24	24	32	32	32	32	31	28	17	17	15	31	32	0
S7P	23	23	24	25	25	25	27	25	25	25	27	27	27	23	24	25	25	23	23	23		24	32	32	32	32	31	28	24	24	24	31	32	0
E4P	23	23	23	23	23	25	27	25	25	25	27	27	27	23	25	25	25	23	23	23	23		32	32	32	32	31	28	24	25	24	31	32	0
ADO	28	28	28	28	28	28	28	28	28	28	29	29	29	28	28	28	28	28	28	28	28	28		9	27	27	32	32	32	32	32	20	32	0
AMP	28	28	28	28	28	28	28	28	28	28	29	29	29	28	28	28	28	28	28	28	28	28	9		16	27	32	32	32	32	32	20	32	0
ADP	28	28	28	28	28	28	28	28	28	28	29	29	29	28	28	28	28	28	28	28	28	28	26	26		24	32	32	32	32	32	32	32	0
ATP	28	28	28	28	28	28	28	28	28	28	29	29	29	28	28	28	28	28	28	28	28	28	27	26	25		32	29	32	32	32	32	32	0
PRPP	28	28	28	28	28	28	28	28	28	28	29	29	29	28	28	29	29	28	28	28	28	28	27	27	27	27		31	31	31	31	31	32	0
IMP	29	29	29	29	29	29	29	29	29	29	29	29	29	29	29	29	29	29	29	29	29	29	29	29	29	29	27		28	31	28	31	32	0
INO	23	23	23	23	23	25	27	25	25	25	27	27	27	20	24	25	25	16	16	16	23	23	28	28	28	28	28	29		17	17	31	32	0
HX	29	29	29	29	29	29	29	29	29	29	29	29	29	29	29	29	29	29	29	29	29	29	29	29	29	29	29	28	29		17	31	32	0
R1P	23	23	23	23	23	25	27	25	25	25	27	27	27	20	24	25	25	15	15	15	23	23	28	28	28	28	28	29	16	29		31	32	0
ADE	27	27	27	27	27	27	27	27	27	27	27	27	27	27	27	27	27	27	27	27	27	27	28	28	28	28	23	28	27	29	27		32	0
NAI	29	29	29	29	29	29	29	29	29	29	30	30	30	29	29	30	30	29	29	29	29	29	29	29	29	29	29	29	29	30	29	29		30
KI	30	30	30	30	30	30	30	30	30	30	30	30	30	30	30	30	30	30	30	30	30	30	30	30	30	30	30	30	30	30	30	30	30	

Supplemental Figure 9

	G6P	F6P	FDP	DHAP	GAP	DPG13	DPG23	PG3	PG2	PEP	PYR	LAC	NADH	GL6P	GO6P	NADPH	GSH	RU5P	R5P	X5P	S7P	E4P	ADO	AMP	ADP	ATP	PRPP	IMP	INO	HX	R1P	ADE	NAI	KI
G6P		4	22	22	22	24	27	24	24	24	22	27	27	26	26	26	26	25	25	25	25	25	30	30	30	30	30	28	25	31	25	30	31	31
F6P	1		22	22	22	24	27	24	24	24	22	27	27	26	26	26	26	25	25	25	25	25	30	30	30	30	30	28	25	31	25	30	31	31
FDP	20	20		14	14	24	27	24	24	24	27	27	27	26	26	26	26	25	25	25	25	25	30	30	30	30	30	28	25	31	25	30	31	31
DHAP	20	20	14		3	24	27	24	24	24	27	27	27	26	26	26	26	25	25	25	25	25	30	30	30	30	30	28	25	31	25	30	31	31
GAP	20	20	14	3		24	27	24	24	24	27	27	27	26	26	26	26	25	25	25	25	25	30	30	30	30	30	28	25	31	25	30	31	31
DPG13	25	25	25	25	25		27	23	23	23	27	22	27	26	26	26	26	25	25	25	25	25	30	30	30	30	30	28	25	31	25	30	31	31
DPG23	27	27	27	27	27	27		27	27	27	27	27	27	27	27	27	27	27	27	27	27	27	31	31	31	31	31	28	27	31	27	31	30	30
PG3	25	25	25	25	25	20	26		8	8	27	23	23	27	27	26	26	27	27	27	27	27	30	30	30	30	30	28	27	31	27	30	31	31
PG2	25	25	25	25	25	20	26	8		5	27	23	23	27	27	26	26	27	27	27	27	27	30	30	30	30	30	28	27	31	27	30	31	31
PEP	25	25	25	25	25	20	26	8	5		27	23	23	27	27	26	26	27	27	27	27	27	30	30	30	30	30	28	27	31	27	30	31	31
PYR	27	27	27	27	27	27	26	27	27	27		23	23	27	27	30	30	27	27	27	27	27	30	30	30	30	30	28	27	30	27	30	31	31
LAC	27	27	27	27	27	27	26	27	27	27	24		23	27	27	30	30	27	27	27	27	27	30	30	30	30	30	28	27	30	27	30	31	31
NADH	27	27	27	27	27	27	26	27	27	27	24	24		27	27	30	30	27	27	27	27	27	30	30	30	30	30	28	27	30	27	30	31	31
GL6P	23	23	24	24	24	25	27	25	25	25	27	27	27		26	26	26	26	26	26	26	26	30	30	30	30	31	28	26	31	26	30	31	31
GO6P	24	24	25	25	25	25	27	25	25	25	27	27	27	24		25	25	26	26	26	26	26	30	30	30	30	31	28	26	31	26	30	31	31
NADPH	25	25	25	25	25	25	26	25	25	25	24	23	24	25	25		1	26	26	26	26	21	30	30	30	30	30	31	26	30	26	30	31	31
GSH	25	25	25	25	25	25	26	25	25	25	24	23	24	25	25	1		26	26	26	26	21	30	30	30	30	30	31	26	30	26	30	31	31
RU5P	23	23	23	23	23	25	27	25	25	25	27	27	27	20	24	25	25		12	7	23	23	30	30	30	30	31	28	16	16	15	31	31	31
R5P	23	23	23	23	23	25	27	25	25	25	27	27	27	20	24	25	25	12		12	23	23	30	30	30	30	31	28	16	16	15	31	31	31
X5P	23	23	23	23	23	25	27	25	25	25	27	27	27	20	24	25	25	7	12		23	23	30	30	30	30	31	28	16	16	15	31	31	31
S7P	23	23	24	25	25	25	27	25	25	25	27	27	27	23	24	25	25	23	23	23		22	31	31	31	31	31	28	23	23	23	31	31	31
E4P	23	23	23	23	23	25	27	25	25	25	27	27	27	23	25	25	25	23	23	23	23		30	30	30	30	31	28	23	24	23	31	31	31
ADO	28	28	28	28	28	28	28	28	28	28	29	29	29	28	28	28	28	28	28	28	28	28		9	26	27	27	31	30	30	30	24	31	31
AMP	28	28	28	28	28	28	28	28	28	28	29	29	29	28	28	28	28	28	28	28	28	28	9		23	27	24	31	30	30	30	23	31	31
ADP	28	28	28	28	28	28	28	28	28	28	29	29	29	28	28	28	28	28	28	28	28	28	26	26		23	23	31	30	30	30	20	31	31
ATP	28	28	28	28	28	28	28	28	28	28	29	29	29	28	28	28	28	28	28	28	28	28	27	26	25		23	31	30	30	30	24	31	31
PRPP	28	28	28	28	28	28	28	28	28	28	29	29	29	28	28	29	29	28	28	28	28	28	27	27	27	27		30	31	30	31	23	31	31
IMP	29	29	29	29	29	29	29	29	29	29	29	29	29	29	29	29	29	29	29	29	29	29	29	29	29	29	27		28	30	28	30	31	31
INO	23	23	23	23	23	25	27	25	25	25	27	27	27	20	24	25	25	16	16	16	23	23	28	28	28	28	28	29		16	16	31	31	31
HX	29	29	29	29	29	29	29	29	29	29	29	29	29	29	29	29	29	29	29	29	29	29	29	29	29	29	29	28	29		16	30	31	31
R1P	23	23	23	23	23	25	27	25	25	25	27	27	27	20	24	25	25	15	15	15	23	23	28	28	28	28	28	29	16	29		31	31	31
ADE	27	27	27	27	27	27	27	27	27	27	27	27	27	27	27	27	27	27	27	27	27	27	28	28	28	28	23	28	27	29	27		31	31
NAI	29	29	29	29	29	29	29	29	29	29	30	30	30	29	29	30	30	29	29	29	29	29	29	29	29	29	29	29	29	30	29	29		30
KI	30	30	30	30	30	30	30	30	30	30	30	30	30	30	30	30	30	30	30	30	30	30	30	30	30	30	30	30	30	30	30	30	30	

Supplemental Figure 10

	G6P	F6P	FDP	DHAP	GAP	DPG13	DPG23	PG3	PG2	PEP	PYR	LAC	NADH	GL6P	GO6P	NADPH	GSH	RU5P	R5P	X5P	S7P	E4P	ADO	AMP	ADP	ATP	PRPP	IMP	INO	HX	R1P	ADE	NAI	KI
G6P		4	24	24	24	24	29	24	24	24	31	31	31	26	26	31	31	25	25	25	25	25	31	31	27	27	29	28	25	29	25	29	32	32
F6P	1		24	24	24	24	29	24	24	24	31	31	31	26	26	31	31	25	25	25	25	25	31	31	27	27	29	28	25	29	25	29	32	32
FDP	20	20		14	14	24	29	24	24	24	31	31	31	26	26	31	31	25	25	25	25	25	31	27	27	27	29	28	25	29	25	29	32	32
DHAP	20	20	14		3	24	29	24	24	24	31	31	31	26	26	31	31	25	25	25	25	25	31	30	27	27	29	28	25	29	25	29	32	32
GAP	20	20	14	3		24	29	24	24	24	31	31	31	26	26	31	31	25	25	25	25	25	31	30	27	27	29	28	25	29	25	29	32	32
DPG13	25	25	25	25	25		29	22	22	22	31	31	31	26	26	31	31	25	25	25	25	25	31	31	30	27	29	28	25	29	25	29	32	32
DPG23	27	27	27	27	27	27		27	27	27	27	27	27	29	29	30	30	29	29	29	29	29	31	31	31	31	30	29	29	30	29	30	32	32
PG3	25	25	25	25	25	20	26		8	8	31	22	17	26	26	30	30	25	25	25	26	26	31	31	31	30	29	28	25	29	25	29	32	32
PG2	25	25	25	25	25	20	26	8		5	31	22	17	26	26	30	30	25	25	25	25	26	31	31	31	30	29	28	25	29	25	29	32	32
PEP	25	25	25	25	25	20	26	8	5		31	22	17	26	26	30	30	25	25	25	25	26	31	31	31	30	29	28	25	29	25	29	32	32
PYR	27	27	27	27	27	27	26	27	27	27		22	22	31	31	30	30	31	31	31	31	31	31	31	31	31	31	31	31	31	31	31	32	32
LAC	27	27	27	27	27	27	26	27	27	27	24		22	31	31	30	30	31	31	31	31	31	31	31	31	31	31	31	31	31	31	31	32	32
NADH	27	27	27	27	27	27	26	27	27	27	24	24		31	31	30	30	31	31	31	31	31	31	31	31	31	31	31	31	31	31	31	32	32
GL6P	23	23	24	24	24	25	27	25	25	25	27	27	27		26	31	31	26	26	26	26	26	31	31	27	27	29	28	26	29	26	29	32	32
GO6P	24	24	25	25	25	25	27	25	25	25	27	27	27	24		31	31	26	26	26	26	26	31	31	27	27	29	28	26	29	26	29	32	32
NADPH	25	25	25	25	25	25	26	25	25	25	24	23	24	25	25		1	31	31	31	31	21	31	31	31	31	30	30	31	30	31	29	32	32
GSH	25	25	25	25	25	25	26	25	25	25	24	23	24	25	25	1		31	31	31	31	21	31	31	31	31	30	30	31	30	31	29	32	32
RU5P	23	23	23	23	23	25	27	25	25	25	27	27	27	20	24	25	25		12	7	23	23	31	31	27	27	29	28	16	16	15	29	32	32
R5P	23	23	23	23	23	25	27	25	25	25	27	27	27	20	24	25	25	12		12	23	23	31	31	30	27	29	28	16	16	15	29	32	32
X5P	23	23	23	23	23	25	27	25	25	25	27	27	27	20	24	25	25	7	12		23	23	31	31	27	27	29	28	16	16	15	29	32	32
S7P	23	23	24	25	25	25	27	25	25	25	27	27	27	23	24	25	25	23	23	23		23	31	31	30	27	29	28	23	27	23	29	32	32
E4P	23	23	23	23	23	25	27	25	25	25	27	27	27	23	25	25	25	23	23	23	23		31	31	29	27	29	28	23	27	23	29	32	32
ADO	28	28	28	28	28	28	28	28	28	28	29	29	29	28	28	28	28	28	28	28	28	28		9	24	27	31	31	31	31	31	31	32	32
AMP	28	28	28	28	28	28	28	28	28	28	29	29	29	28	28	28	28	28	28	28	28	28	9		23	24	31	31	31	31	31	31	32	32
ADP	28	28	28	28	28	28	28	28	28	28	29	29	29	28	28	28	28	28	28	28	28	28	26	26		23	31	31	27	31	30	31	32	32
ATP	28	28	28	28	28	28	28	28	28	28	29	29	29	28	28	28	28	28	28	28	28	28	27	26	25		31	31	27	31	27	31	32	32
PRPP	28	28	28	28	28	28	28	28	28	28	29	29	29	28	28	29	29	28	28	28	28	28	27	27	27	27		29	29	27	29	21	32	32
IMP	29	29	29	29	29	29	29	29	29	29	29	29	29	29	29	29	29	29	29	29	29	29	29	29	29	29	27		28	29	28	29	32	32
INO	23	23	23	23	23	25	27	25	25	25	27	27	27	20	24	25	25	16	16	16	23	23	28	28	28	28	28	29		16	16	29	32	32
HX	29	29	29	29	29	29	29	29	29	29	29	29	29	29	29	29	29	29	29	29	29	29	29	29	29	29	29	28	29		16	27	32	32
R1P	23	23	23	23	23	25	27	25	25	25	27	27	27	20	24	25	25	15	15	15	23	23	28	28	28	28	28	29	16	29		29	32	32
ADE	27	27	27	27	27	27	27	27	27	27	27	27	27	27	27	27	27	27	27	27	27	27	28	28	28	28	23	28	27	29	27		32	32
NAI	29	29	29	29	29	29	29	29	29	29	30	30	30	29	29	30	30	29	29	29	29	29	29	29	29	29	29	29	29	30	29	29		30
KI	30	30	30	30	30	30	30	30	30	30	30	30	30	30	30	30	30	30	30	30	30	30	30	30	30	30	30	30	30	30	30	30	30	

Supplemental Figure 11

	G6P	F6P	FDP	DHAP	GAP	DPG13	DPG23	PG3	PG2	PEP	PYR	LAC	NADH	GL6P	GO6P	NADPH	GSH	RU5P	R5P	X5P	S7P	E4P	ADO	AMP	ADP	ATP	PRPP	IMP	INO	HX	R1P	ADE	NAI	KI
G6P		4	24	24	24	24	29	24	24	24	31	31	31	26	26	31	31	25	25	25	25	25	31	31	27	27	29	28	25	29	25	29	32	32
F6P	1		24	24	24	24	29	24	24	24	31	31	31	26	26	31	31	25	25	25	25	25	31	31	27	27	29	28	25	29	25	29	32	32
FDP	20	20		14	14	24	29	24	24	24	31	31	31	26	26	31	31	25	25	25	25	25	31	27	27	27	29	28	25	29	25	29	32	32
DHAP	20	20	14		3	24	29	24	24	24	31	31	31	26	26	31	31	25	25	25	25	25	31	27	27	27	29	28	25	29	25	29	32	32
GAP	20	20	14	3		24	29	24	24	24	31	31	31	26	26	31	31	25	25	25	25	25	31	27	27	27	29	28	25	29	25	29	32	32
DPG13	25	25	25	25	25		29	22	22	22	31	31	31	26	26	31	31	25	25	25	25	25	31	31	30	27	29	28	25	29	25	29	32	32
DPG23	27	27	27	27	27	27		27	27	27	27	27	27	29	29	30	30	29	29	29	29	29	31	31	31	31	30	29	29	30	29	30	32	32
PG3	25	25	25	25	25	20	26		8	8	31	22	17	26	26	30	30	25	25	25	26	26	31	31	31	30	29	28	25	29	25	29	32	32
PG2	25	25	25	25	25	20	26	8		5	31	22	17	26	26	30	30	25	25	25	26	26	31	31	31	30	29	28	25	29	25	29	32	32
PEP	25	25	25	25	25	20	26	8	5		31	22	17	26	26	30	30	25	25	25	26	26	31	31	31	30	29	28	25	29	25	29	32	32
PYR	27	27	27	27	27	27	26	27	27	27		22	22	31	31	30	30	31	31	31	31	31	31	31	31	31	31	31	31	31	31	31	32	32
LAC	27	27	27	27	27	27	26	27	27	27	24		22	31	31	30	30	31	31	31	31	31	31	31	31	31	31	31	31	31	31	31	32	32
NADH	27	27	27	27	27	27	26	27	27	27	24	24		31	31	30	30	31	31	31	31	31	31	31	31	31	31	31	31	31	31	31	32	32
GL6P	23	23	24	24	24	25	27	25	25	25	27	27	27		26	31	31	26	26	26	26	26	31	31	27	27	29	28	26	29	26	29	32	32
GO6P	24	24	25	25	25	25	27	25	25	25	27	27	27	24		31	31	26	26	26	26	26	31	31	27	27	29	28	26	29	26	29	32	32
NADPH	25	25	25	25	25	25	26	25	25	25	24	23	24	25	25		1	31	31	31	31	21	31	31	31	31	30	30	31	30	31	29	32	32
GSH	25	25	25	25	25	25	26	25	25	25	24	23	24	25	25	1		31	31	31	31	21	31	31	31	31	30	30	31	30	31	29	32	32
RU5P	23	23	23	23	23	25	27	25	25	25	27	27	27	20	24	25	25		12	7	23	23	31	31	27	27	29	28	16	16	15	29	32	32
R5P	23	23	23	23	23	25	27	25	25	25	27	27	27	20	24	25	25	12		12	23	23	31	31	27	27	29	28	16	16	15	29	32	32
X5P	23	23	23	23	23	25	27	25	25	25	27	27	27	20	24	25	25	7	12		23	23	31	31	27	27	29	28	16	16	15	29	32	32
S7P	23	23	24	25	25	25	27	25	25	25	27	27	27	23	24	25	25	23	23	23		23	31	31	30	27	29	28	23	27	23	29	32	32
E4P	23	23	23	23	23	25	27	25	25	25	27	27	27	23	25	25	25	23	23	23	23		31	31	27	27	29	28	23	27	23	29	32	32
ADO	28	28	28	28	28	28	28	28	28	28	29	29	29	28	28	28	28	28	28	28	28	28		9	24	27	31	31	31	31	31	31	32	32
AMP	28	28	28	28	28	28	28	28	28	28	29	29	29	28	28	28	28	28	28	28	28	28	9		23	24	31	31	31	31	31	31	32	32
ADP	28	28	28	28	28	28	28	28	28	28	29	29	29	28	28	28	28	28	28	28	28	28	26	26		23	31	31	27	31	27	31	32	32
ATP	28	28	28	28	28	28	28	28	28	28	29	29	29	28	28	28	28	28	28	28	28	28	27	26	25		31	31	27	31	27	31	32	32
PRPP	28	28	28	28	28	28	28	28	28	28	29	29	29	28	28	29	29	28	28	28	28	28	27	27	27	27		29	29	27	29	21	32	32
IMP	29	29	29	29	29	29	29	29	29	29	29	29	29	29	29	29	29	29	29	29	29	29	29	29	29	29	27		28	29	28	29	32	32
INO	23	23	23	23	23	25	27	25	25	25	27	27	27	20	24	25	25	16	16	16	23	23	28	28	28	28	28	29		16	16	29	32	32
HX	29	29	29	29	29	29	29	29	29	29	29	29	29	29	29	29	29	29	29	29	29	29	29	29	29	29	29	28	29		16	27	32	32
R1P	23	23	23	23	23	25	27	25	25	25	27	27	27	20	24	25	25	15	15	15	23	23	28	28	28	28	28	29	16	29		29	32	32
ADE	27	27	27	27	27	27	27	27	27	27	27	27	27	27	27	27	27	27	27	27	27	27	28	28	28	28	23	28	27	29	27		32	32
NAI	29	29	29	29	29	29	29	29	29	29	30	30	30	29	29	30	30	29	29	29	29	29	29	29	29	29	29	29	29	30	29	29		30
KI	30	30	30	30	30	30	30	30	30	30	30	30	30	30	30	30	30	30	30	30	30	30	30	30	30	30	30	30	30	30	30	30	30	

Supplemental Figure 12

	G6P	F6P	FDP	DHAP	GAP	DPG13	DPG23	PG3	PG2	PEP	PYR	LAC	NADH	GL6P	GO6P	NADPH	GSH	RU5P	R5P	X5P	S7P	E4P	ADO	AMP	ADP	ATP	PRPP	IMP	INO	HX	R1P	ADE	NAI	KI
G6P		4	24	24	24	24	27	27	27	27	28	28	28	26	26	26	26	25	25	25	25	25	29	29	29	29	31	28	25	25	25	29	31	31
F6P	1		24	24	24	24	27	27	27	27	28	28	28	26	26	26	26	25	25	25	25	25	29	29	29	29	31	28	25	25	25	29	31	31
FDP	20	20		14	14	24	27	27	27	27	30	30	30	26	26	26	26	25	25	25	25	25	17	29	29	29	31	28	25	25	25	29	31	31
DHAP	20	20	14		3	24	27	27	27	27	30	30	30	26	26	26	26	25	25	25	25	25	14	29	29	29	31	28	25	25	25	29	31	31
GAP	20	20	14	3		24	27	27	27	27	30	30	30	26	26	26	26	25	25	25	25	25	14	29	29	29	31	28	25	25	25	29	31	31
DPG13	25	25	25	25	25		27	22	22	22	27	23	23	26	26	26	26	25	25	25	25	25	29	29	29	29	31	28	25	29	25	29	31	31
DPG23	27	27	27	27	27	27		27	27	27	27	27	27	27	27	27	27	27	27	27	27	27	29	29	29	29	31	28	27	29	27	29	31	31
PG3	25	25	25	25	25	20	26		8	8	27	22	17	27	27	27	27	27	27	27	27	27	29	29	29	29	31	28	27	29	27	29	31	31
PG2	25	25	25	25	25	20	26	8		5	27	22	17	27	27	27	27	27	27	27	27	27	29	29	29	29	31	28	27	29	27	29	31	31
PEP	25	25	25	25	25	20	26	8	5		27	22	17	27	27	27	27	27	27	27	27	27	29	29	29	29	31	28	27	29	27	29	31	31
PYR	27	27	27	27	27	27	26	27	27	27		22	22	27	27	27	27	27	27	27	27	27	30	30	30	30	31	28	27	30	27	30	31	31
LAC	27	27	27	27	27	27	26	27	27	27	24		22	27	27	27	27	27	27	27	27	27	30	30	30	30	31	28	27	30	27	30	31	31
NADH	27	27	27	27	27	27	26	27	27	27	24	24		27	27	27	27	27	27	27	27	27	30	30	30	30	31	28	27	30	27	30	31	31
GL6P	23	23	24	24	24	25	27	25	25	25	27	27	27		26	26	26	26	26	26	26	26	29	29	29	29	31	28	26	26	26	29	31	31
GO6P	24	24	25	25	25	25	27	25	25	25	27	27	27	24		26	26	26	26	26	26	26	29	29	29	29	31	28	26	26	26	29	31	31
NADPH	25	25	25	25	25	25	26	25	25	25	24	23	24	25	25		1	26	26	26	26	21	29	29	29	29	31	28	26	26	26	29	31	31
GSH	25	25	25	25	25	25	26	25	25	25	24	23	24	25	25	1		26	26	26	26	21	29	29	29	29	31	28	26	26	26	29	31	31
RU5P	23	23	23	23	23	25	27	25	25	25	27	27	27	20	24	25	25		12	7	23	24	29	29	29	29	31	28	16	16	15	29	31	31
R5P	23	23	23	23	23	25	27	25	25	25	27	27	27	20	24	25	25	12		12	23	24	29	29	29	29	31	28	16	16	15	29	31	31
X5P	23	23	23	23	23	25	27	25	25	25	27	27	27	20	24	25	25	7	12		23	24	29	29	29	29	31	28	16	16	15	29	31	31
S7P	23	23	24	25	25	25	27	25	25	25	27	27	27	23	24	25	25	23	23	23		24	29	29	29	29	31	28	22	23	22	29	31	31
E4P	23	23	23	23	23	25	27	25	25	25	27	27	27	23	25	25	25	23	23	23	23		29	29	29	29	31	28	24	24	24	29	31	31
ADO	28	28	28	28	28	28	28	28	28	28	29	29	29	28	28	28	28	28	28	28	28	28		9	27	27	31	29	29	29	29	20	31	31
AMP	28	28	28	28	28	28	28	28	28	28	29	29	29	28	28	28	28	28	28	28	28	28	9		17	27	31	29	29	29	29	20	31	31
ADP	28	28	28	28	28	28	28	28	28	28	29	29	29	28	28	28	28	28	28	28	28	28	26	26		24	31	29	29	29	29	24	31	31
ATP	28	28	28	28	28	28	28	28	28	28	29	29	29	28	28	28	28	28	28	28	28	28	27	26	25		31	29	29	29	29	27	31	31
PRPP	28	28	28	28	28	28	28	28	28	28	29	29	29	28	28	29	29	28	28	28	28	28	27	27	27	27		31	31	31	31	31	30	30
IMP	29	29	29	29	29	29	29	29	29	29	29	29	29	29	29	29	29	29	29	29	29	29	29	29	29	29	27		28	29	28	29	31	31
INO	23	23	23	23	23	25	27	25	25	25	27	27	27	20	24	25	25	16	16	16	23	23	28	28	28	28	28	29		16	16	29	31	31
HX	29	29	29	29	29	29	29	29	29	29	29	29	29	29	29	29	29	29	29	29	29	29	29	29	29	29	29	28	29		16	29	31	31
R1P	23	23	23	23	23	25	27	25	25	25	27	27	27	20	24	25	25	15	15	15	23	23	28	28	28	28	28	29	16	29		29	31	31
ADE	27	27	27	27	27	27	27	27	27	27	27	27	27	27	27	27	27	27	27	27	27	27	28	28	28	28	23	28	27	29	27		31	31
NAI	29	29	29	29	29	29	29	29	29	29	30	30	30	29	29	30	30	29	29	29	29	29	29	29	29	29	29	29	29	30	29	29		30
KI	30	30	30	30	30	30	30	30	30	30	30	30	30	30	30	30	30	30	30	30	30	30	30	30	30	30	30	30	30	30	30	30	30	

Supplemental Figure 13

	G6P	F6P	FDP	DHAP	GAP	DPG13	DPG23	PG3	PG2	PEP	PYR	LAC	NADH	GL6P	GO6P	NADPH	GSH	RU5P	R5P	X5P	S7P	E4P	ADO	AMP	ADP	ATP	PRPP	IMP	INO	HX	R1P	ADE	NAI	KI
G6P		4	24	24	24	24	27	24	24	24	27	27	27	26	26	26	26	25	25	25	25	25	29	29	29	29	30	28	25	25	25	29	30	30
F6P	1		24	24	24	24	27	24	24	24	27	27	27	26	26	26	26	25	25	25	25	25	29	29	29	29	30	28	25	25	25	29	30	30
FDP	20	20		14	14	24	27	24	24	24	27	27	27	26	26	26	26	25	25	25	25	25	17	29	29	29	30	28	25	25	25	29	30	30
DHAP	20	20	14		3	24	27	24	24	24	27	27	27	26	26	26	26	25	25	25	25	25	17	29	29	29	30	28	25	25	25	29	30	30
GAP	20	20	14	3		24	27	24	24	24	27	27	27	26	26	26	26	25	25	25	25	25	17	29	29	29	30	28	25	25	25	29	30	30
DPG13	25	25	25	25	25		27	22	22	22	27	23	23	26	26	26	26	25	25	25	25	25	29	29	29	29	27	28	25	28	25	29	30	30
DPG23	27	27	27	27	27	27		27	27	27	27	27	27	27	27	27	27	27	27	27	27	27	29	29	29	29	1	28	27	29	27	29	30	30
PG3	25	25	25	25	25	20	26		8	8	27	22	17	27	27	26	26	27	27	27	27	27	29	29	29	29	27	28	27	29	27	29	30	30
PG2	25	25	25	25	25	20	26	8		5	27	22	17	27	27	26	26	27	27	27	27	27	29	29	29	29	27	28	27	29	27	29	30	30
PEP	25	25	25	25	25	20	26	8	5		27	22	17	27	27	26	26	27	27	27	27	27	29	29	29	29	27	28	27	29	27	29	30	30
PYR	27	27	27	27	27	27	26	27	27	27		22	22	27	27	27	27	27	27	27	27	27	29	29	29	29	27	28	27	29	27	29	30	30
LAC	27	27	27	27	27	27	26	27	27	27	24		22	27	27	27	27	27	27	27	27	27	29	29	29	29	27	28	27	29	27	29	30	30
NADH	27	27	27	27	27	27	26	27	27	27	24	24		27	27	27	27	27	27	27	27	27	29	29	29	29	27	28	27	29	27	29	30	30
GL6P	23	23	24	24	24	25	27	25	25	25	27	27	27		26	26	26	26	26	26	26	26	29	29	29	29	30	28	26	26	26	29	30	30
GO6P	24	24	25	25	25	25	27	25	25	25	27	27	27	24		25	25	26	26	26	26	26	29	29	29	29	30	28	26	26	26	29	30	30
NADPH	25	25	25	25	25	25	26	25	25	25	24	23	24	25	25		1	26	26	26	26	21	29	29	29	29	27	28	26	26	26	29	30	30
GSH	25	25	25	25	25	25	26	25	25	25	24	23	24	25	25	1		26	26	26	26	21	29	29	29	29	27	28	26	26	26	29	30	30
RU5P	23	23	23	23	23	25	27	25	25	25	27	27	27	20	24	25	25		12	7	23	23	29	29	29	29	30	28	16	16	15	29	30	30
R5P	23	23	23	23	23	25	27	25	25	25	27	27	27	20	24	25	25	12		12	23	23	29	29	29	29	30	28	16	16	15	29	30	30
X5P	23	23	23	23	23	25	27	25	25	25	27	27	27	20	24	25	25	7	12		23	23	29	29	29	29	30	28	16	16	15	29	30	30
S7P	23	23	24	25	25	25	27	25	25	25	27	27	27	23	24	25	25	23	23	23		23	29	29	29	29	30	28	23	23	23	29	30	30
E4P	23	23	23	23	23	25	27	25	25	25	27	27	27	23	25	25	25	23	23	23	23		29	29	29	29	30	28	23	24	23	29	30	30
ADO	28	28	28	28	28	28	28	28	28	28	29	29	29	28	28	28	28	28	28	28	28	28		9	27	27	30	29	29	29	29	20	30	30
AMP	28	28	28	28	28	28	28	28	28	28	29	29	29	28	28	28	28	28	28	28	28	28	9		23	27	30	29	29	29	29	20	30	30
ADP	28	28	28	28	28	28	28	28	28	28	29	29	29	28	28	28	28	28	28	28	28	28	26	26		24	30	29	29	29	29	24	30	30
ATP	28	28	28	28	28	28	28	28	28	28	29	29	29	28	28	28	28	28	28	28	28	28	27	26	25		30	29	29	29	29	27	30	30
PRPP	28	28	28	28	28	28	28	28	28	28	29	29	29	28	28	29	29	28	28	28	28	28	27	27	27	27		28	30	30	30	30	29	29
IMP	29	29	29	29	29	29	29	29	29	29	29	29	29	29	29	29	29	29	29	29	29	29	29	29	29	29	27		28	29	28	29	30	30
INO	23	23	23	23	23	25	27	25	25	25	27	27	27	20	24	25	25	16	16	16	23	23	28	28	28	28	28	29		16	16	29	30	30
HX	29	29	29	29	29	29	29	29	29	29	29	29	29	29	29	29	29	29	29	29	29	29	29	29	29	29	29	28	29		16	28	30	30
R1P	23	23	23	23	23	25	27	25	25	25	27	27	27	20	24	25	25	15	15	15	23	23	28	28	28	28	28	29	16	29		29	30	30
ADE	27	27	27	27	27	27	27	27	27	27	27	27	27	27	27	27	27	27	27	27	27	27	28	28	28	28	23	28	27	29	27		30	30
NAI	29	29	29	29	29	29	29	29	29	29	30	30	30	29	29	30	30	29	29	29	29	29	29	29	29	29	29	29	29	30	29	29		1
KI	30	30	30	30	30	30	30	30	30	30	30	30	30	30	30	30	30	30	30	30	30	30	30	30	30	30	30	30	30	30	30	30	30	

Supplemental Figure 14

	G6P	F6P	FDP	DHAP	GAP	DPG13	DPG23	PG3	PG2	PEP	PYR	LAC	NADH	GL6P	GO6P	NADPH	GSH	RU5P	R5P	X5P	S7P	E4P	ADO	AMP	ADP	ATP	PRPP	IMP	INO	HX	R1P	ADE	NAI	KI
G6P		4	22	22	22	24	27	24	24	24	22	27	27	26	26	26	26	25	25	25	25	25	31	31	31	31	31	28	25	31	25	31	31	31
F6P	1		22	22	22	24	27	24	24	24	22	27	27	26	26	26	26	25	25	25	25	25	31	31	31	31	31	28	25	31	25	31	31	31
FDP	20	20		14	14	24	27	24	24	24	27	27	27	26	26	26	26	25	25	25	25	25	31	31	31	31	31	28	25	31	25	31	31	31
DHAP	20	20	14		3	24	27	24	24	24	27	27	27	26	26	26	26	25	25	25	25	25	31	31	31	31	31	28	25	31	25	31	31	31
GAP	20	20	14	3		24	27	24	24	24	27	27	27	26	26	26	26	25	25	25	25	25	31	31	31	31	31	28	25	31	25	31	31	31
DPG13	25	25	25	25	25		27	23	23	23	27	22	27	26	26	26	26	25	25	25	25	25	31	31	31	31	31	28	25	31	25	31	31	31
DPG23	27	27	27	27	27	27		27	27	27	27	27	27	27	27	30	30	27	27	27	27	27	30	30	30	30	30	28	27	30	27	30	31	31
PG3	25	25	25	25	25	20	26		8	8	27	23	23	27	27	26	26	27	27	27	27	27	31	31	31	31	31	28	27	31	27	31	30	30
PG2	25	25	25	25	25	20	26	8		5	27	23	23	27	27	26	26	27	27	27	27	27	31	31	31	31	31	28	27	31	27	31	30	30
PEP	25	25	25	25	25	20	26	8	5		27	23	23	27	27	26	26	27	27	27	27	27	31	31	31	31	31	28	27	31	27	31	30	30
PYR	27	27	27	27	27	27	26	27	27	27		23	23	27	27	30	30	27	27	27	27	27	30	30	30	30	30	28	27	30	27	30	31	31
LAC	27	27	27	27	27	27	26	27	27	27	24		23	27	27	30	30	27	27	27	27	27	30	30	30	30	30	28	27	30	27	30	31	31
NADH	27	27	27	27	27	27	26	27	27	27	24	24		27	27	30	30	27	27	27	27	27	30	30	30	30	30	28	27	30	27	30	31	31
GL6P	23	23	24	24	24	25	27	25	25	25	27	27	27		26	26	26	26	26	26	26	26	31	31	31	31	31	28	26	31	26	31	30	30
GO6P	24	24	25	25	25	25	27	25	25	25	27	27	27	24		25	25	26	26	26	26	26	31	31	31	31	31	28	26	31	26	31	30	30
NADPH	25	25	25	25	25	25	26	25	25	25	24	23	24	25	25		1	26	26	26	26	21	30	30	30	30	30	30	26	30	26	30	31	31
GSH	25	25	25	25	25	25	26	25	25	25	24	23	24	25	25	1		26	26	26	26	21	30	30	30	30	30	30	26	30	26	30	31	31
RU5P	23	23	23	23	23	25	27	25	25	25	27	27	27	20	24	25	25		12	7	23	23	31	31	31	31	31	28	16	16	15	31	31	31
R5P	23	23	23	23	23	25	27	25	25	25	27	27	27	20	24	25	25	12		12	23	23	31	31	31	31	31	28	16	16	15	31	31	31
X5P	23	23	23	23	23	25	27	25	25	25	27	27	27	20	24	25	25	7	12		23	23	31	31	31	31	31	28	16	16	15	31	31	31
S7P	23	23	24	25	25	25	27	25	25	25	27	27	27	23	24	25	25	23	23	23		22	31	31	31	31	31	28	23	23	23	31	31	31
E4P	23	23	23	23	23	25	27	25	25	25	27	27	27	23	25	25	25	23	23	23	23		31	31	31	31	31	28	23	31	23	31	31	31
ADO	28	28	28	28	28	28	28	28	28	28	29	29	29	28	28	28	28	28	28	28	28	28		9	26	27	27	30	31	30	31	24	31	31
AMP	28	28	28	28	28	28	28	28	28	28	29	29	29	28	28	28	28	28	28	28	28	28	9		23	27	24	30	31	30	31	23	31	31
ADP	28	28	28	28	28	28	28	28	28	28	29	29	29	28	28	28	28	28	28	28	28	28	26	26		23	23	30	31	30	31	20	31	31
ATP	28	28	28	28	28	28	28	28	28	28	29	29	29	28	28	28	28	28	28	28	28	28	27	26	25		23	30	31	30	31	23	31	31
PRPP	28	28	28	28	28	28	28	28	28	28	29	29	29	28	28	29	29	28	28	28	28	28	27	27	27	27		30	31	30	31	22	31	31
IMP	29	29	29	29	29	29	29	29	29	29	29	29	29	29	29	29	29	29	29	29	29	29	29	29	29	29	27		28	30	28	30	31	31
INO	23	23	23	23	23	25	27	25	25	25	27	27	27	20	24	25	25	16	16	16	23	23	28	28	28	28	28	29		16	16	31	31	31
HX	29	29	29	29	29	29	29	29	29	29	29	29	29	29	29	29	29	29	29	29	29	29	29	29	29	29	29	28	29		16	30	31	31
R1P	23	23	23	23	23	25	27	25	25	25	27	27	27	20	24	25	25	15	15	15	23	23	28	28	28	28	28	29	16	29		31	31	31
ADE	27	27	27	27	27	27	27	27	27	27	27	27	27	27	27	27	27	27	27	27	27	27	28	28	28	28	23	28	27	29	27		31	31
NAI	29	29	29	29	29	29	29	29	29	29	30	30	30	29	29	30	30	29	29	29	29	29	29	29	29	29	29	29	29	30	29	29		30
KI	30	30	30	30	30	30	30	30	30	30	30	30	30	30	30	30	30	30	30	30	30	30	30	30	30	30	30	30	30	30	30	30	30	

Supplemental Figure 15

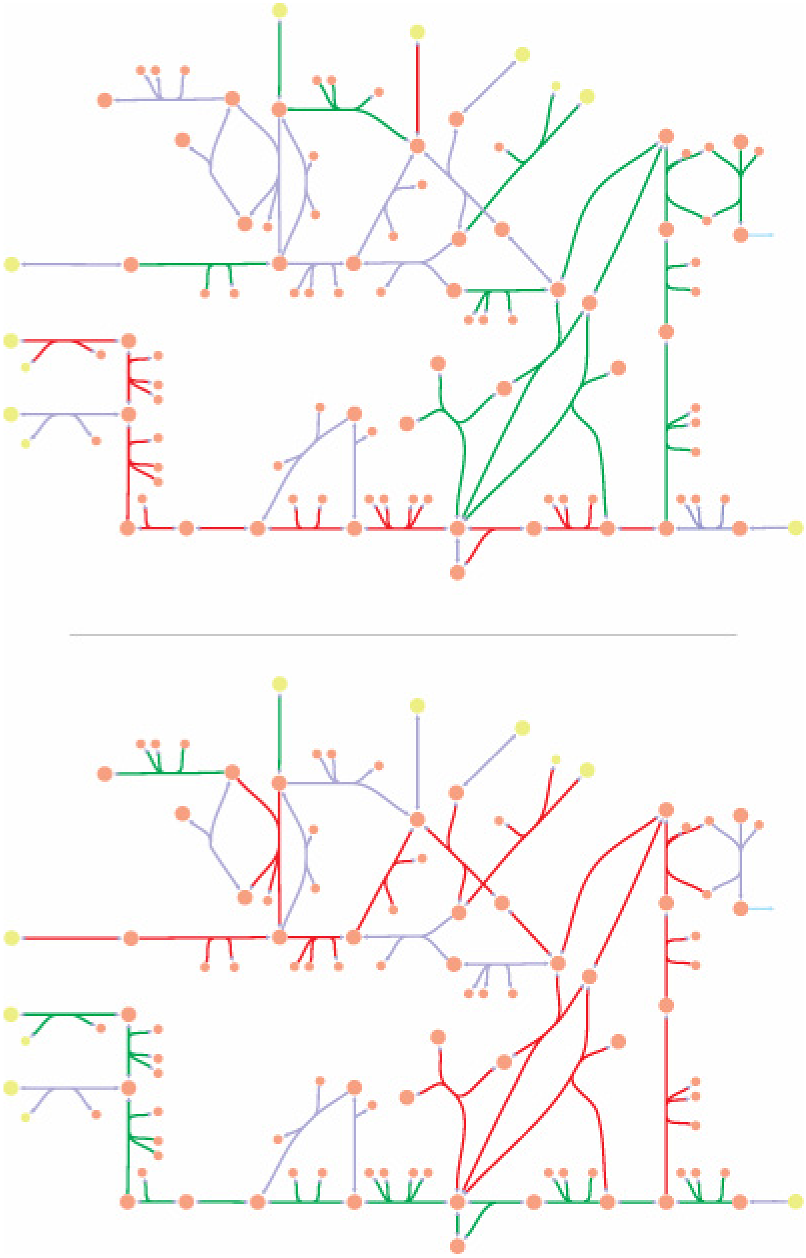

Supplemental Figure 16

Supplemental Figure Legends

Supplemental Figure 1: Comparison of aggregate pool formation of metabolites under a high redox load state compared to a low redox load state for a 'normal' human erythrocyte. For each condition, the time scales at which the metabolites pooled with one another (cutoff 90% of maximum for the normed modal matrix) was determined. The numbers in the lower left triangle indicate the time scale (lower numbers correspond to faster time scales) after which the two corresponding metabolites pool together for the lower redox state. The upper right triangular entries indicate the same but for the cell at the high redox state. Changes in two or three time scales generally does not indicate a meaningful difference because many of the intermediate time scales are not well separated and the motions of the metabolite interactions between these modes often overlap.
The grayscale shading indicates whether metabolites pool on different time scales under the different conditions (white indicates no difference, i.e. pooling occurs at the same time scale and gray indicates large differences in pooling time scales). The diagonal entries are always blackened. A completely white plot would indicate that there is no difference in the hierarchical structure between the two conditions.
A perfectly symmetrical plot would indicate that dynamically there are not any differences in terms of pooling; thus increased assymetries reflected disruptions in the pooling between metabolites. The shifts in pooling time scales are observed throughout the network, however the most pronounced shifts involve the interactions between GL6P and the hexose-phosphates, the pentose phosphates and glutathione/NADPH, and the high energy phosphates (AMP, ADP, ATP) with the nucleotide precursor and degradation products.

Supplemental Figure 2: Hierarchical correlations between metabolites were made as described in the text, for the A+ G6PD variant (non-chronic hemolytic anemia) under high redox loads and summarized in the upper right triangle of the plot. The lower left triangle is the normal red cell under a high redox load. Other details about the plot are as described in Figure 1. The plot has very few gray/shaded areas, indicating high similarity to the normal cell when responding to high redox loads.

Supplemental Figure 3: Comparison between a high load and low load for the A- G6PD variant (non-chronic hemolytic anemia). The details and organization of the plot are the same as those found in Figure 6. A high degree of similarity between the A- and normal erythrocyte under high redox load conditions is observed.

Supplemental Figure 4-7: Comparison between a high redox loaded G6PD variants: Iwate, Niigata, Yamaguchi, and Portici (upper right triangles) respectively, with the normal red cell at a high redox load (lower left triangle). All of these represent patients with chronic hemolytic anemia. The details and organization of the plot are the same as those described for Figure 1. The largest difference involve interactions between the oxidative branch of the pentose phosphate pathway and the first half of glycolysis, interactions among members of the non-oxidative branch of the pentose phosphate pathway, and the nucleotide salvage pathway metabolites.

Supplemental Figure 8: Comparison between the high energy load state and low energy load state for a 'normal' human erythrocyte. An analogous format and organization to Figure 1 is shown. There are relatively small changes in the pooling structure that are pervasive throughout the network. The most significant changes in pooling time scale among metabolites involves: ADO and AMP with FDP, DHAP, and GAP; HX with RU5P, X5P and R1P, and S7P; ADP with AMP.

Supplemental Figure 9-15: Comparison between the PK variants: Mantova, Brescia, Soresina, Sassari, Sassari 2, Parma, and Milano at high energy loads (upper right triangles) respectively with the normal red cell at a high energy load (lower left triangles). These variants all exhibit fairly similar patterns. The observed shading patterns are similar to the shaded regions observed in Figure 8. The salient observations to be made from these plots are 1) the variants all exhibit fairly similar patterns of variation (unlike the G6PD variants) and 2) the asymmetrical patterns in Figures 9-15 mirror the changes observed in Figure 8, i.e. the comparison between the normal red cell at two different energy loads.

Supplemental Figure 16: Illustration of general changes in fluxes in response to redox (upper panel) or energy (lower panel) loads for the nRBC. Green shading reflects increases in the fluxes relative to the unstressed case and red shading reflects decreases.
